# Neuroprotective Effects of Chemical Constituents of Leaves of *Euonymus hamiltonianus* Wall.

**DOI:** 10.3390/plants13081094

**Published:** 2024-04-13

**Authors:** Huynh Nguyen Khanh Tran, Jae Sik Yu, Tianqi Huang, Gakyung Lee, Hyo Sun Choi, Hyun Ok Yang

**Affiliations:** 1 Natural Products Research Center, Korea Institute of Science and Technology, Gangneung 25451, Republic of Korea; hnktran2404@gmail.com (H.N.K.T.); 618509@kist.re.kr (T.H.);; 2 Department of Integrative Biological Sciences and Industry & Convergence Research Center for Natural Products, Sejong University, 209 Neungdong-ro, Gwangjin-gu, Seoul 05006, Republic of Korea; jsyu@sejong.ac.kr (J.S.Y.); lgg1025@sejong.ac.kr (G.L.)

**Keywords:** *Euonymus hamiltonianus*, flavonols, beta amyloid, NO, iNOS, COX-2

## Abstract

*Euonymus hamiltonianus* Wall. is considered a medicinal plant and is used to treat pain, cough, dysuria, and cancer, but a clear phytochemical investigation of its biological activities has yet to be performed. Investigation of chemical constituents of the leaves of *Euonymus hamiltonianus* Wall. led to the isolation of three new compounds by chromatography techniques, euonymusins A–C (**1**, **10**, and **11**), and the acquisition of new spectroscopic data for euonymusin D (**2**), along with the identification of ten known compounds. The chemical structures of the compounds were established using extensive spectroscopic techniques, including NMR, MS, and hydrolysis, and compared with the published data. These compounds were tested in vitro for their inhibitory effects on beta amyloid production (Aβ42). Compounds **13** and **14** displayed weak inhibition, with IC_50_ values ranging from 53.15 to 65.43 µM. Moreover, these compounds were also assessed for their inhibitory effects on nitric oxide production. Of these compounds, **3**, **4**, and **14** displayed inhibitory effects on NO production, with IC_50_ values ranging from 14.38 to 17.44 µM. Compounds **3**, **4**, and **14** also suppressed LPS-induced expression of nitric oxide synthase (iNOS) and cyclooxygenase-2 (COX-2) protein.

## 1. Introduction

Neurodegenerative diseases, such as Alzheimer’s disease (AD), Parkinson’s disease (PD), and multiple sclerosis (MS), affect the lives of millions of patients across the world and cause a variety of socio-economic problems [1]. The main reasons for these symptoms are the formation and accumulation of extracellular senile plaques of amyloid beta (Aβ) [2,3]. It was reported that Aβ is the main component and plays an important role in AD expression [4], causing astrocyte activation and neuronal apoptosis, leading to the deterioration of AD. Aβ is produced by proteolysis of amyloid precursor protein (APP), a type I transmembrane protein. APP can be cleaved in mediated stages through amyloidogenic pathways by β-secretase 1 (BACE1) and then γ-secretase [5]. Therefore, inhibition of the aggregation of Aβ peptides is considered one of the potential pieces of evidence for the neuroprotective effect of natural products in the progression of neurodegenerative diseases [6]. Additionally, during this progress, microglial cells are a type of CNS immune cell which play an essential role in immune defense and tissue repair with a self-limiting mechanism. Over-activation of microglia has been determined as a property of neurodegenerative diseases, leading to the production of various proinflammatory cytokines and neurotoxic substances, including interleukin-1β (IL-1β), IL-6, tumor necrosis factor-alpha (TNF-α), and nitric oxide (NO) [1]. NO is a product of the inducible isoforms of inducible nitric oxide synthase (iNOS) and cyclooxygenase (COX-2) enzymes. It was demonstrated that iNOS is not normally expressed in the brain, but LPS upregulates iNOS expression in microglial cells, astrocytes, and possibly neurons [7]. Two forms of COX, COX-1 and COX-2, are known to be induced in response to many stimulants and to be activated in sites of inflammation [8]. Several studies have reported that COX-2 is associated with cytotoxicity in neurodegenerative diseases. Taken together, the inflammatory mediators, including iNOS and COX-2, are responsible for the symptoms of severe neuronal damage in neurodegenerative diseases [7]. 

Phytochemical compounds, originating from plant sources, have demonstrated promising capabilities in the treatment of neurodegenerative conditions [9]. As more and more evidence suggests that chronic inflammation caused by microglia contributes to disorders in the central nervous system, it is becoming clear that anti-inflammatory natural products and their components can serve as powerful protectors against a range of CNS pathologies [10]. *Euonymus hamiltonianus* Wall. is a medicinal plant belonging to the Celastraceae family, which is distributed in many Asian countries, including Korea, Japan, China, and India [9]. This indigenous plant is a deciduous tree that has been used for traditional treatment of various conditions, such as diuretic, carminative, stimulant, gynecological, coughing, pain, and cancer-related diseases [10]. This medicinal plant species, *E. hamiltonianus*, was used to treat pain, cough, dysuria, and cancer and exhibited effects of enhanced memory and cognitive abilities when applied as a functional food in the prevention and treatment of cognitive impairment-related diseases [11,12]. The genus *Euonymus* of the family Celastraceae has been investigated and commonly compared to the species *E. hamiltonianus*. The genus Euonymus is reported to contain sesquiterpenes, flavonoids, and coumarins [13,14,15]. As of this moment, there are only a limited number of reports on the phytochemistry and related activities performed by *E. hamiltonianus*. Previous studies have shown some of its phytochemicals by identifying novel coumarins in this indigenous plant, but these phytochemicals did not perform any related bioactivity [16]. Furthermore, to date, only three triterpenoids, six sesquiterpenoids, two flavonoids, and two coumarins have been identified in this plant species, and further in-depth phytochemical investigation of *E. hamiltonianus* is still needed [17,18,19,20]. 

As part of an ongoing chemical investigation to discover new potential drug candidates derived from natural products for the treatment of neurodegenerative diseases [7], 30% ethanol extraction was selected for further research due to the strong inhibition observed in total processed extractions with different ratios of ethanol and water. This prompted us to identify the active principles of *E. hamiltonianus* by bioactivity-guided fractionation [Figure S1]. This study describes the isolation, structural elucidation, and biological investigation of three new compounds, the acquisition of new spectroscopic data for one compound, and the identification of ten known compounds in relation to the inhibition of Aβ peptide production in two kinds of cells. The products whose inhibition was assessed were Aβ42 isoforms in APPsw-transfected HeLa cells and proinflammatory factor secretions, such as NO, iNOS, and COX-2, in lipopolysaccharide (LPS)-activated BV-2 microglial cells.

## 2. Results and Discussion

### 2.1. Structural Determination of Compounds ***1***, ***2***, ***10***, and ***11***

**Figure 1:**
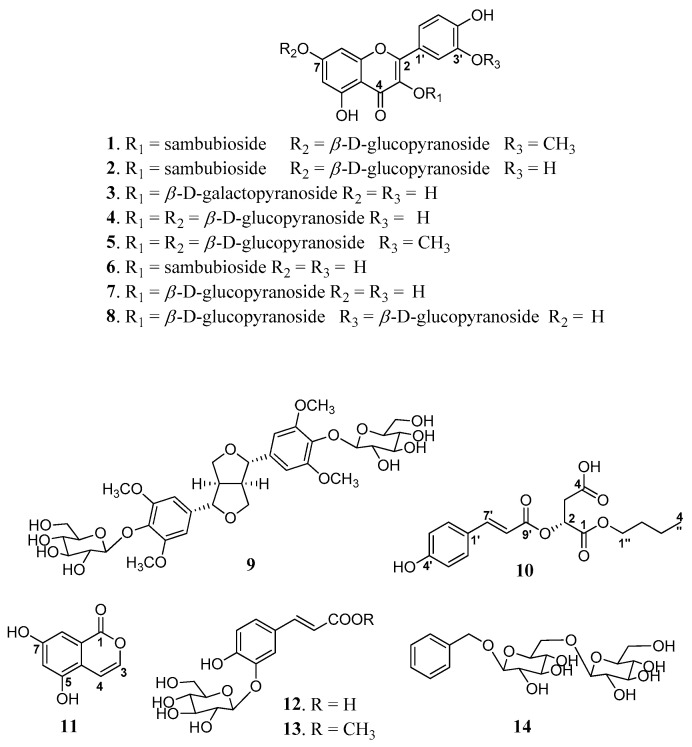
The chemical structures of compounds (**1**–**14**) isolated from the leaves of *E. hamiltonianus* Wall.

**Figure 2:**
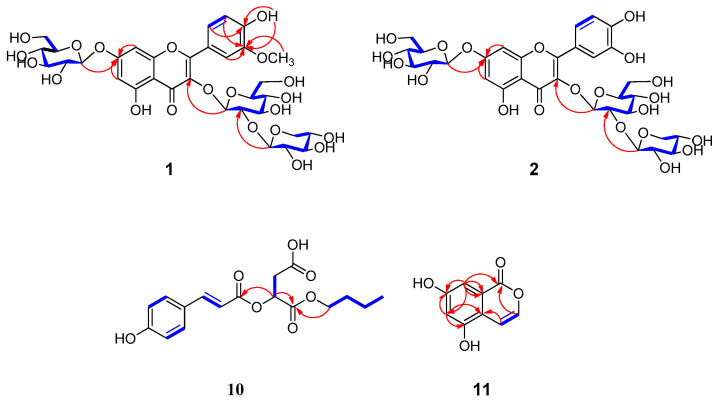
The selected ^1^H-^1^H COSY (blue bold lines) and HMBC (red arrow lines) correlations of the new compounds **1**, **2**, **10**, and **11**.

Compound **1** was a yellowish, amorphous powder, and its molecular formula of C_33_H_40_O_21_ was determined by high-resolution electrospray ionization mass spectrometry (HR-ESIMS) with *m*/*z* 773.2138 [M + H]^+^ (calcd. for C_33_H_41_O_21_, 773.2135) (Figure 1). Its IR spectrum showed the presence of hydroxyl (3266 cm^−1^), carbonyl (1732 cm^−1^), and aromatic ring (1471 cm^−1^) absorption bands, while the UV spectrum suggested the presence of a highly conjugated system at 250 nm. The 1H NMR spectrum of 1 indicated the existence of an ABX system at *δ*_H_ 7.86 (1H, d, *J* = 2.0 Hz, H-2′), 7.65 (1H, d, *J* = 8.5, 2.0 Hz, H-6′), and 6.93 (1H, d, *J* = 8.5 Hz, H-5′); a pair of meta-coupled aromatic protons, *δ*_H_ 6.82 (1H, d, *J* = 2.0 Hz, H-8) and 6.44 (1H, d, *J* = 2.0 Hz, H-6); three β-anomeric protons, *δ*_H_ 5.76 (1H, d, *J* = 7.0 Hz, H-1″), 5.08 (1H, d, *J* = 7.5 Hz, H-1⁗), and 4.62 (1H, d, *J* = 7.5 Hz, H-1‴); and one methoxy group, *δ*_H_ 3.85 (3H, s, 3′-OCH_3_). The ^13^C NMR spectrum of **1** showed 33 signals that indicated the presence of two benzene rings, a methoxy group (*δ*_C_ 55.8), and one ketone group (*δ*_C_ 177.6), as well as three anomeric carbon units representing sugar moieties, two glucose units (*δ*_C_ 98.1 and 99.8) and one xylose unit (*δ*_C_ 104.2). The spectroscopic data of **1** showed a close resemblance to the flavonoid glycoside isorhamnetin-3,7-*O*-di-β-d-glucopyranoside that was isolated from *Brassica rapa* [21]. Both were closely related structures, but compound **1** had an additional xylose group that was positioned at C-2″ in **1**. This was further confirmed by a heteronuclear multiple bond correlation (HMBC) experiment, showing a significant correlation between *δ*_H_ 4.62 (Xylose, H-1‴) and *δ*_C_ 81.6 (C-2″) (Figure 2). Acid hydrolysis of 1 by 5% HCl/MeOH confirmed the presence of D-glucose and xylose, which were determined by comparisons of the retention times with those of authentic samples (Figure S6) [22]. Therefore, **1** was identified to be isorhamnet-in-3-*O*-(2″-*O*-(β-d-xylopyranosyl)-β-d-glucopyranoside)-7-*O*-β-d-glucopyranoside (Figure 1). It was named euonymusin A.

Compound **2** was obtained as a yellowish, amorphous powder. HR-ESIMS data showed a molecular ion peak at *m*/*z* 759.2010 [M + H]^+^ (calcd. for C_32_H_39_O_21_, 759.1978), indicating a molecular formula of C_32_H_38_O_21_. Its IR spectrum showed the presence of hydroxyl (3309 cm^−1^), carbonyl (1731 cm^−1^), and aromatic ring (1454 cm^−1^) absorption bands. The UV spectrum suggested the appearance of the highest absorption band at 250 nm. The spectroscopic data of **2** closely resembled those of **1** (Table 1), except for the fact the latter had one less methoxy group. **2** was previously identified as quercetin-3-*O*-(2″-*O*-(β-d-xylopyranosyl)-β-d-glucopyranoside)-7-*O*-β-d-glucopyranoside by the personal empirical experience of Fiasson, K. G. and co-workers without significant spectroscopic data to support the identification (Figure 1) [23]. This study reports the first full spectroscopic data for a purified compound, **2** (Figure S3.1–S3.6). Therefore, the structure of **2** was completely determined, and it was named euonymusin D.

Compound **10** was obtained as a yellow, amorphous powder and displayed the adduct ion in the HR-ESIMS data at *m*/*z* 359.1092 [M + Na]^+^ (calcd. for C_17_H_20_O_7_Na, 359.1101). The ^1^H NMR spectrum (Table 1) of 10 indicated the characteristic signals of an AA’BB’ system at *δ*_H_ 7.59 (d, *J* = 8.5 Hz) and 6.79 (d, *J* = 8.5 Hz) and olefinic signals at *δ*_H_ 7.60 (d, *J* = 16.0 Hz) and 6.46 (d, *J* = 16.0 Hz). The presence of additional carbonyl and methylene was also deduced from the signals at *δ*_H_ 5.37 (dd, *J* = 8.0, 4.0 Hz), 2.86 (dd, *J* = 12.0, 4.5 Hz), and 2.79 (dd, *J* = 12.0, 3.5 Hz). Moreover, the signal of an *n*-butyl moiety was distributed at *δ*_H_ 4.10, 1.54, 1.30 (each 2H, m), and 0.86 (t, *J* = 7.0 Hz). The ^13^C NMR spectroscopic data of 10 indicated the presence of three carbonyl groups at 170.7, 169.0, and 165.8. Overall, the NMR spectrum showed a close resemblance to previously isolated phytochemical 2-*O*-(*trans*-coumaroyl)malic acid [24]. Taken together, compound **10** appeared to possess a coumaroyl moiety in the *trans-*configuration, malic acid, and an *n*-butyl moiety in its structure. Compound **10** has an additional butyl moiety attached, as was determined via HMBC, according to which H-1″ (*δ*_H_ 4.10) correlated with C-1 (*δ*_C_ 169.0). Other HMBC correlations are mentioned in Figure 2. With this evidence, compound **10** was determined as *p*-coumaroylmalic acid 1-butyl ester. The configuration of **10** was determined by comparison of specific optical rotation values with reported values. The optical rotation value of 10 {$a_{D}^{23}$-27.3 (c 1.1, MeOH)} was in good agreement with that of p-coumaroyl-d-malic acid 1-methyl ester {$a_{D}^{25}$-12.8 (c 0.5, MeOH)}, whereas (+)-(*E*)-caffeoyl-l-malic acid had an opposite value of {$a_{D}^{20}$+31.5 (c 1.36, H_2_O)} [25]. Thus, the structure of **10** was determined (Figure 1), and it was named euonymusin B.

Compound **11** was obtained as a colorless, amorphous powder. Its molecular formula was determined to be C_9_H_6_O_4_ by HR-ESIMS at *m*/*z* 179.0343 [M + H]^+^ (calcd. for C_9_H_7_O_4_, 179.0344). Its IR spectrum showed the presence of hydroxyl (3440 cm^−1^), carbonyl (1777 cm^−1^), and aromatic ring (1370 cm^−1^) absorption bands. Its UV spectrum showed maximum absorption at 250 nm. The ^1^H NMR spectrum of **11** exhibited signals of a pair of *cis*-coupled protons, *δ*_H_ 7.97 (1H, d, *J* = 5.6 Hz, H-3) and 6.19 (1H, ovl, H-4), and a pair of *meta*-coupled protons, *δ*_H_ 6.33 (1H, d, *J* = 1.6 Hz, H-8) and 6.19 (1H, ovl, H-6). The ^13^C NMR spectrum showed nine carbon signals, comprising one benzene ring, two olefinic groups, and one carbonyl group (*δ*_C_ 183.4). The above results indicated that compound **11** was an isocoumarin derivative similar to 5,7-dihydroxy-4-methylisocoumarin [26], but compound **11** did not have an additional methyl group. The position of functional groups was also clarified by HBMC correlations (Figure 2). Thus, the structure of **11** was conclusively determined to be 5,7-dihydroxyisocoumarin (Figure 1), and it was named euonymusin C.

Repeated column chromatography of *n*-BuOH soluble fractions led to the isolation of three new compounds, euonymusin A–D (**1**, **10**, and **11**); one new set of spectroscopic data that had not previously been reported (**2**); together with ten known compounds whose chemical structures were identified as those of known compounds by comparing their spectroscopic data with data reported in the literature, namely, quercetin-3-*O*-β-d-galactopyranoside (**3**) [27], quercetin-3,7-di-*O*-β-d-glucopyranoside (**4**) [27], isorhamnetin-3,7-di-*O*-β-d-glucopyranoside (**5**) [28], quercetin-3-*O*-sambubioside (**6**) [29], isoquercetin (**7**) [30], quercetin-3,3′-di-*O*-β-d-glucopyranoside (**8**) [31], liriodendrin (**9**) [32], caffeic acid *trans*-3-*O*-β-d-glucopyranoside (**12**) [33], methyl *trans*-3-*O*-(β-d-glucopyranosyl) caffeate (**13**) [34], and benzyl gentiobioside (**14**) [34] (Figure 1).

### 2.2. Chemical Profiling of Phytochemicals ***1***–***14***

**Figure 3:**
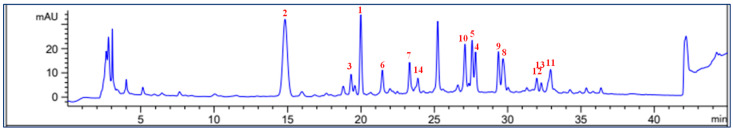
The representative HPLC profile of the main peaks (**1**–**14**) derived from 30% EtOH extract of the leaves of *E. hamiltonianus* Wall. obtained with a PDA detector at 254 nm and a YMC-Triart C18 ExRS column (4.6 mm × 250 mm, 5 μm particle size). The solvent system used for the chromatogram was as follows: 0–10 min (ACN/H_2_O = 10/90), 10–25 min (10–20%), 25–38 min (20–30%), 38–40 min (30–100%), 40–45 min (100%).

In this study, qualitative profile analysis was performed on the 30% EtOH crude extract of the leaves of *E. hamiltonianus* using HPLC. The isolated phytochemicals, **1**–**14**, were used as standards and compared to the crude extract profile represented by a high-performance liquid chromatogram (Figure 3). While analyzing the chemical profile of the HPLC chromatogram, the isolated compound **2** showed signs of degradation. With comparative UV area normalization, compound **2** was confirmed as a pure compound initially when isolated (Figure S3.8). After compound **2** was stored for 27 days, the compound was re-evaluated through LC-MS analysis and showed signs of degradation (Figure S3.9). For reconfirmation, LC-MS analyses of both the dried powdered form of the compound when isolated and the compound in DMSO solution were performed (Figure S3.9), both sets of results showing signs of degradation. Compound **2** was degraded into three separate peaks, and the original compound **2** indicated by *m*/*z* 759.2 was verified by LC-MS analysis, and the peaks of **2a** and **2b** were indicated by *m*/*z* 465.1 and 627, respectively. This suggested that the original compound **2** was partially degraded into different quercetin glycosides, with possible hydrolysis of the sugar moieties. Due to the close resemblance of compound **2** to compound **1**, it was investigated in the same manner after a significant length of time. Compound **1** did not show any sign of degradation (Figure S3.10), and the structural difference between **1** and **2** is the substitution of a methoxy group at C-3′. Previous studies suggested that the presence of abundant hydroxyl groups in the chemical structures of flavonoids can facilitate degradation, whereas substituents of sugar moieties and methoxyl groups can possibly protect against degradation of flavonoids [35,36].

### 2.3. Inhibition of the Aβ Aggregation of Compound ***13***

Preventing the harmful aggregation of Aβ peptides has been indicated to be a promising strategy against neurodegenerative disease [37]. Phytomedicines have been widely reported to be utilized in the treatment of neurodegenerative diseases and other conditions associated with Aβ [38]. The total extraction and fractions (Figure S1) of *E. hamiltonianus* displayed inhibitory effects against Aβ production [19]. Secretion levels of Aβ42 were significantly decreased by EA and BuOH fractions, with inhibitions of 50.6% and 50.4% at 25 µg/mL, respectively. All isolated compounds were tested for their inhibitory activity against Aβ42 production in APPsw-transfected HeLa cells. Among them, compound **13** showed weak inhibition of Aβ42 production with an IC_50_ value of 65.43 µM (Table 2).

The accumulation of Aβ is a characteristic trait of both hereditary and random occurrences of AD. The intense focus of pharmaceutical developments for the treatment of AD is to decrease Aβ levels, a task that seems to be quite difficult [39]. Inhibitors that have been directly proven to be effective in reducing Aβ production in the lab have encountered potential issues during clinical trials [40]. This study shows that only compound **13** (Table 2) reduced Aβ levels in Hela-swAPP cells. This suggests that while compound **13** may not be highly potent or efficient, it does interact with Aβ in a way that results in inhibition. The inability to detect decreased Aβ in the supernatants of Hela cells could be due to the extremely low amounts of the peptide these cells secrete. Considering the relative content in the extract, the effect was similar between the subfraction Bu6 + 7 (Figure S1) and compound **13**, and this indicates that **13** may possibly alleviate AD via targeting anti-β-amyloidosis during the pathogenesis of AD.

### 2.4. Anti-Inflammation Effect of the Isolated Compounds ***3***, ***4***, and ***14***

**Figure 4:**
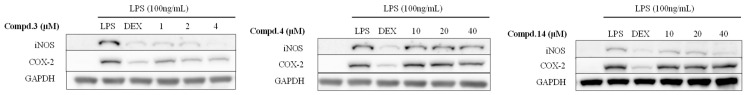
Inhibition of LPS-induced iNOS and COX-2 expression in BV-2 microglial cells by **3**, **4**, and **14**. Respective Western blots presenting the expression level of iNOS and COX-2. Each group of experiments was repeated three times (n = 3).

The cytotoxic effects of the isolated compounds (**1**–**14**) were evaluated by the MTT assay and did not affect the viability of BV-2 microglial cells following 12 h of treatment in the presence or absence of LPS, even at a concentration of 100 µM (Table 2). The anti-inflammatory effects of the fourteen compounds (**1**–**14**) isolated from *E. hamiltonianus* were evaluated in activated BV-2 microglial cells by the production of NO and proinflammatory cytokines. BV-2 microglial cells were stimulated with 0.1 µg/mL LPS for 12 h in the presence of the compounds at various concentrations, and the levels of NO in the culture supernatants were measured using Griess reactions. Dexamethasone was used as a positive control compound, with an IC_50_ value of 1.24 µM. The results of the NO production assay, including IC_50_ values, are summarized in Table 2. Among them, those for compounds **3**, **4**, and **14** are displayed, their IC_50_ values ranging from 14.38 to 17.44 µM, but the other compounds were inactive. In a comparison of the activities of the pair of **4** (IC_50_ = 14.38 µM) and **5** (IC_50_ > 100 µM), the substituted hydroxy group was indicated to have a more potent activity against NO production than methoxy substitution at position C-3′. In the same manner, the substituted sugar moiety at C-3 also showed a different inhibition of NO production compared with **3** (galactose, IC_50_ = 14.38 µM) and **6** and **7** (sambubiose and glucose, respectively; IC_50_ > 100 µM), showing greater potential inhibition for the substituted galactopyranoside unit. The compounds were continuously assessed to determine whether they suppressed the expression of iNOS and COX-2 in LPS-stimulated BV-2 microglial cells. The cells were stimulated with LPS in the presence of these compounds at various concentrations, and the expression levels of iNOS and COX-2 were determined by Western blotting. The results indicated that **3**, **4**, and **14** also downregulated expression levels of LPS-induced iNOS and COX-2 proteins in a concentration-dependent manner (Figure 4). Among them, compound **3** exhibited the most potent inhibitory activity against LPS-induced iNOS and COX-**2** expression at treatment concentrations of 1.0, 2.0, and 4.0 µM. Although compound **3** inhibited NO production to a somewhat lesser extent than compound **4**, **3** significantly suppressed iNOS and COX-2 protein expression compared to **4**. Thus, compound **3** may be considered a potential anti-inflammatory agent. 

Lipopolysaccharide (LPS), a key constituent of the cell wall in Gram-negative bacteria, is instrumental in triggering inflammation, with the ability to activate immune cells and provoke an inflammatory reaction [41]. And microglia residing in the CNS plays a crucial role in preserving the balance within its microenvironment. When microglia are activated by LPS, they release a large number of cytotoxic mediators, such as NO, causing neuronal damage [42]. Phytochemicals, which are secondary metabolites produced by plants, serve as a defense mechanism against environmental pressures like LPS-induced inflammatory stress [43]. The treatments with compounds **3**, **4**, and **14** effectively downregulated the production of inflammatory mediators, including NO and iNOS, against LPS. Moreover, some research reports have found that certain phytochemicals can inhibit the production of NO and iNOS without inhibiting COX-2 [44]. In our study, a similar phenomenon was observed in the treatment of compound **14**, which showed no significant effect in terms of COX-2 inhibition. It was indicated that induced COX-2 can mobilize excessive release of PGE2, which can be a great booster for the progress of inflammation [45]. Therefore, inhibition of COX-2 will also be an essential effect when investigating the anti-inflammatory activity of natural products. Combined with iNOS and NO inhibition, compounds **3** and **4** showed significant anti-inflammatory activity, and compound 3 in particular can be a potential target for potential development as an anti-inflammation constituent of *E. hamiltonianus*.

## 3. Materials and Methods

### 3.1. General Experimental Procedures

UV spectra were recorded using a Thermo spectrometer. IR spectra were recorded using a JASCO FT/IR-4100 spectrometer (JASCO, Easton, MD, USA). The 1D- and 2D-NMR spectra were obtained using Varian Unity Inova 400 MHz and 500 MHz spectrometers with tetramethylsilane (TMS) as an internal standard, and the chemical shifts were recorded as δ values (ppm). Mass spectra were recorded using a Thermo Scientific™ (Q Exactive) EASY-nLC 1000 spectrometer (San Jose, CA, USA). Silica gel (Merck, Darmstadt, Germany; 63–200 µm particle size), RP-C_18_ (Merck; 75 µm particle size), sephadex LH-20 (Pharmacia, Uppsala, Sweden ), and dianion HP-20 (Supelco, St. Louis, MI, USA) were used for column chromatography. TLC was performed using Merck silica gel 60 F254 and RP-C_18_ F254 plates (Merck, Darmstadt, Germany). Preparative high-performance liquid chromatography (HPLC) was performed using a Water System with a UV detector 2996 (Waters, Miford, MA, USA) and a YMC-Triart C18 column (10 mm × 250 mm, 5 μm particle size; YMC Co., Ltd., Kyoto, Japan). Compounds were visualized after spraying with aqueous 10% H_2_SO_4_ and heating for 3–5 min.

### 3.2. Plant Materials

The leaves of *E. hamiltonianus* Wall. were collected at Hantaek Botanical Garden (37.094376, 127.406739; entrance of the botanical garden) in Yongin-si, Gyeonggi-do, Republic of Korea, from August to October 2019. Botanical identification was performed by Prof. Hyun Ok Yang, and the voucher specimen KGC07EH was deposited at the Herbarium of the Korea Institute of Science and Technology (Gangneung, Gangwon-do, Republic of Korea).

### 3.3. Extraction and Isolation

The leaves of *E. hamiltonianus* Wall. (3.0 kg) were extracted three times (3 h × 15 L) with 30% EtOH. After the solvent was removed under reduced pressure, the residue was suspended in H_2_O and then partitioned with *n*-hexane (0.5 g), EtOAc (12.0 g), *n*-BuOH (136.0 g), and residue, successively. The BuOH-soluble fraction was continuously chromatographed (diaion HP-20, Supelco) on a silica gel column using a stepwise gradient of H_2_O:MeOH (100%:0 to 0:100%, each 20 L) to yield thirteen fractions (EHBu.1–13), while the EtOAc-soluble fraction was eluted on a normal phase silica gel column using a solvent gradient of MC:MeOH 40:1 to 0:1 to obtain ten subfractions (EHEA.1–10), according to their TLC profiles. A combined fraction, EHBu.6 + 7 (6.5 g), was subsequently subjected to silica gel column chromatography (60 cm× 6.5 cm) and eluted with MC:MeOH:H_2_O (25:1:0.1) to yield fourteen subfractions (EHBu.6A to N). Subfraction EHBu.6C (1.5 g) was chromatographed on a silica gel column (60 cm × 3.5 cm) using a gradient solvent system of EA:MeOH:H_2_O (8:1:0.1) to obtain 5 subfractions EHBu.6C1–5). Subfractions EHBu.6C2 (950.0 mg) and 6C4 (720.0 mg) were further purified over YMC RP-18, using a sephadex LH-20 column with MeOH-H_2_O (1:1) and a semi-preparative Waters HPLC system with an isocratic solvent system of 30% MeOH in H_2_O + 0.1% formic acid (flow rate 3 mL/min) kept for over 60 min as an eluent to yield **1** (19.0 mg), **2** (55.0 mg), **3** (2.1 mg), **4** (8.0 mg), **5** (3.0 mg), **6** (2.3 mg), **7** (14.0 mg), **8** (2.2 mg), **9** (5.6 mg), **12** (5.0 mg), and **14** (6.0 mg), with UV detection at 210 and 254 nm. In the same manner, compounds **10** (2.4 mg) and **11** (3.8 mg) were isolated from subfractions of EHEA.5 (260.0 mg) and EHEA.8 (140.0 mg), respectively. 

### 3.4. Euonymusin A (***1***)

A yellowish, amorphous powder; UV λ_max_ (DMSO) (ε): 250 (1500) and 300 (1150) nm; IR (ATR) ν_max_: 3266, 1732, 1471, and 1189 cm^−1^; ^1^H and ^13^C NMR (DMSO-*d*_6_) data, see Table 1; HR-ESIMS *m*/*z* 773.2138 [M + H]^+^ (calcd. for C_33_H_41_O_21_, 773.2135).

### 3.5. Euonymusin D (***2***)

A yellowish, amorphous powder; UV λ_max_ (DMSO) (ε): 250 (46) and 300 (35) nm; IR (ATR) ν_max_: 3309, 1731, 1454, and 1196 cm^−1^; ^1^H and ^13^C NMR (DMSO-*d*_6_) data, see Table 1; HR-ESIMS *m*/*z* 759.2010 [M + H]^+^ (calcd. for C_32_H_39_O_21_, 759.1978).

### 3.6. Euonymusin B (***10***)

A yellow, amorphous powder; $a_{D}^{23}$–27.3 (c 1.1, MeOH), UV λ_max_ (DMSO) (ε): 210 (90), 235 (80), and 330 (140) nm; IR (ATR) ν_max_: 3324, 2943, 2838, 1646, 1445, 1413, and 1021 cm^−1^; ^1^H and ^13^C NMR (DMSO-*d*_6_) data, see Table 1; HR-ESIMS *m*/*z* 359.1092 [M + Na]^+^ (calcd. for C_17_H_20_O_7_Na, 359.1101).

### 3.7. Euonymusin C (***11***)

A colorless, amorphous powder; UV λ_max_ (DMSO) (ε): 220 (50), 250 (100), and 290 (40) nm; IR (ATR) ν_max_: 3440, 1777, 1370, and 1193 cm^−1^; ^1^H and ^13^C NMR (methanol-*d*_4_) data, see Table 1; HR-ESIMS *m*/*z* 179.0343 [M + H]^+^ (calcd. for C_9_H_7_O_4_, 179.0344).

### 3.8. Hydrolysis of ***1***

Compound **1** (1.0 mg) was hydrolyzed by 4.0 mL of 5% HCl/MeOH for 3 h at 95 °C. After the reaction, the mixture was dried and separated in one part CH_2_Cl_2_ to three parts water (15 mL CH_2_Cl_2_), after which the water residue was evaporated in vacuo to dryness with MeOH, then analyzed by HPLC, and the results were compared with authentic samples [20].

### 3.9. Aβ Assay

APPsw-transfected HeLa cells were cultured with the isolated compounds or DMSO in Dulbecco’s modified Eagle medium (DMEM, Welgene, Gyeongsan-si, Republic of Korea) for 8 h, and then the cell culture supernatants were collected for the following analyses. For the detection of Aβ secretion, kits for Aβ42 (KHB3442) and Aβ40 (KHB3482) were purchased from the Invitrogen Company and used according to the supplier’s instructions [46].

### 3.10. Cell Cultures and MTT Assay for Cell Viability

For the Aβ-Hela-APPsw model, Hela cells were stably transfected with an APP carrying the Swedish mutation (APPsw) using BioT (Bioland Scientific LLC, Paramount, CA, USA) according to the manufacturer’s instructions, which was provided by Prof. Tae-Wan Kim (Department of Pathology, Columbia University of Medical Center, New York, NY 10032, USA). APPsw-transfected HeLa cells were grown in DMEM supplemented with 10% fetal bovine serum (FBS; Thermo Fisher Scientific, Lafayette, CA, USA), 1% penicillin/streptomycin (Thermo Fisher Scientific, Lafayette, CA, USA), 260 μg/mL Zeocin, and 400 μg/mL G418. The cells were maintained at 37 °C under a humidified atmosphere of 5% CO_2_. The stock sample solution was dissolved in DMSO and kept at −20 °C and diluted to the final concentration in fresh medium before each experiment. For the cell viability assay, Hela cells were cultured in 96-well plates for 24 h and then various concentrations of comp 27 (0–10 μM) were treated for 12 h. The EZ-Cytox kit (DAEILLAB, Cheongwon, Republic of Korea) was applied to detect the cell viability, following the manufacturer’s instructions, and measurements were taken by a microplate reader (BIO-TEK^®^ Dower-Wave; BioTek, Winooski, VT, USA) under absorbance at 450 nm. The results were presented as the percentage of MTT reduction, compared with the control group as 100%. For the LPS-induced microglia model, the BV-2 mouse microglia cell line was provided by Prof. Lee Sung Jung’s research team in the College of Medicine, Seoul National University, and was cultured in DMEM-F12 with 5% FBS and 1% penicillin/streptomycin at 37 °C under a humidified atmosphere containing 5% CO_2_. And the cell viability assay was performed using the Aβ-Hela-APPsw model. 

### 3.11. Determination of NO Production and the Cell Viability Assay

Cells were grown in 6-well plates (5 × 10^5^ cells per well) for 24 h, and after incubation for 24 h, the cells were pre-administrated 5, 10, and 20 µM concentrations of the sample for 1 h, then treated with 0.1 µg/mL LPS for 12 h. The media were collected and centrifuged at 13,000 rpm for the removal of dead cells. The supernatants were collected and mixed with an equal volume (50 μL) of Griess reagents (1% sulfanilamide and N-(1-naphthyl) ethylenediamine dihydrochloride in 2.5% H_3_PO_4_) for 5 min at room temperature, and the Nitric Oxide concentration was measured at 540 nm using a microplate reader and compared with the sodium nitrite standard curve.

### 3.12. Western Blot Analysis

The total protein samples (10 μg) were loaded in sodium dodecyl sulfate polyacrylamide gel electrophoresis (SDS–PAGE) gels with 10% acrylamide/bis and transferred to polyvinylidene difluoride membranes (Millipore, Burlington, MA, USA). Then, the membranes were blocked with 5% silky milk dissolved in TBST (Tris Buffered saline Tween) for 1 h under room temperature and incubated overnight at 4 °C with specific primary antibodies (1:1000). After being washed with TBST, the membranes were incubated with secondary HRP-conjugated IgG (1:2000) at room temperature for 1 h and visualized using enhanced chemiluminescence (ECL) reagents (Thermo Fisher Scientific, Lafayette, CA, USA). Densitometry analysis of the bands was performed with the fusion solo system (Vilber, Paris, France). The band densities were determined as the intensities of protein by TotalLab TL120 software v2009 (TotalLab, Newcastle, UK). 

### 3.13. Statistical Analysis

Data were expressed as the mean ± SD of each independent repeat. For comparison of three or more repeats, data were analyzed by one-way analysis of variance (ANOVA) with Dunnett’s post hoc test. A value of *p* < 0.05 was considered statistically significant. Statistical tests were carried out using GraphPad Prism 5.0 (GraphPad Software, San Diego, CA, USA).

## 4. Conclusions

The chemical investigation of the leaves of *E. hamiltonianus* was carried out and brought a general phytochemical view with a structural characterization of eight flavonols (**1**–**8**), one lignan (**9**), and five phenols (**10**–**14**, including one isocoumarin and four phenolic derivatives). Among them, one novel flavonoid glycoside (**1**), one phenolic acid (**10**), and one coumarin (**11**) were identified as new compounds, and this is the first study to present the purified spectroscopic data of euonymusin D (**2**). The neuroprotective effects of the isolated compounds were elucidated via assessment of their inhibition of Aβ and NO production. Their inhibitory effects were weak with respect to Aβ production, whereas compounds **3**, **4**, and **14** displayed good inhibitory effects against NO production. Furthermore, these compounds suppressed the expression levels of iNOS and COX-2 in LPS-stimulated BV-2 microglial cells. This also suggests that the isolated compounds **3**, **4**, and **14** may be a possible target for future neuroprotective agents. Among published studies, this is the first to report an investigation into the relationship between secondary metabolites obtained from *E. hamiltonianus* species and their bioactivity.

## Figures and Tables

**Table 1:** ^1^H NMR and ^13^C NMR data of compounds **1**, **2**, **10**, and **11**.

Position	1 ^a^		2 ^a^		10 ^a^		11 ^b^	
	*δ*_H_ (*J* in Hz)	*δ*_C_	*δ*_H_ (*J* in Hz)	*δ*_C_	*δ*_H_ (*J* in Hz)	*δ*_C_	*δ*_H_ (*J* in Hz)	*δ*_C_
1						169.0		183.4
2		156.4		156.1	5.37, dd (8.0, 4.0)	67.9		
3a		133.2		133.3	2.86, dd (12.0, 4.5)	35.7	7.97, d (5.6)	158.2
3b					2.79, dd (12.0, 3.5)			
4		177.6		177.6		170.7	6.19, ovl ^c^	111.6
4a		105.7		105.6				159.9
5		160.9		161.0				163.5
6	6.44, d (2.0)	99.3	6.42, d (2.0)	99.3			6.19, ovl ^c^	100.2
7		162.9		162.8				166.2
8	6.82, d (2.0)	94.6	6.76, d (2.0)	94.3			6.33, d (1.6)	95.1
8a		156.0		156.0				106.6
1′		121.0		121.1		124.9		
2′	7.86, d (2.0)	113.2	7.59, d (2.5)	115.3	7.59, d (8.5)	130.5		
3′		147.1		145.0	6.79, d (8.5)	115.5		
4′		149.8		148.8		160.3		
5′	6.93, d (8.5)	115.3	6.86, d (8.0)	116.4	6.79, d (8.5)	115.5		
6′	7.65, dd (8.5, 2.0)	122.8	7.67, dd (8.0, 2.5)	122.1	7.59, d (8.5)	130.5		
7′					7.60, d (16.0)	145.9		
8′					6.46, d (16.0)	112.8		
9′						165.8		
1″	5.76, d (7.0)	98.1	5.73, d (7.0)	97.9	4.10, m	64.4		
2″	3.48, d (7.0)	81.6	3.49, d (7.0)	82.0	1.54, m	29.7		
3″	3.13, m	77.6	3.10, m	77.8	1.30, m	18.2		
4″	3.05, dd (7.0, 3.0)	73.9	3.29, d (8.0)	74.1	0.86, t (7.0)	13.3		
5″	3.25, m	73.2	3.14, m	73.2				
6″a	3.55, m	60.5	3.70, m	60.7				
6″b	3.35, m		3.53, d (11.5)					
1‴	4.62, d (7.5)	104.2	4.58, d (7.5)	104.7				
2‴	3.44, d (8.0)	77.3	3.06, d (7.5)	77.2				
3‴	3.00, d (8.0)	76.2	3.14, m	76.3				
4‴	3.23, m	69.4	3.24, d (9.0)	69.4				
5‴a	3.66, dd (11.0, 5.0)	65.6	3.67, m	65.8				
5‴b	2.98, d (11.0)		3.03, m					
1⁗	5.08, d (7.5)	99.8	5.10, d (7.5)	99.7				
2⁗	3.12, m	69.6	3.27, d (7.5)	69.6				
3⁗	3.28, m	76.5	3.10, m	76.5				
4⁗	3.11, m	77.1	3.46, d (8.0)	76.9				
5⁗	3.16, m	69.5	3.11, m	69.5				
6⁗a	3.69, m	60.7	3.44, m	60.7				
6⁗b	3.46, d (7.0)		3.31, m					
3′-OCH_3_	3.85, s	55.8						
5-OH	12.60, s		12.69, brs					
4′-OH	9.95, brs							
3″-OH	4.46, d (5.5)							
4″-OH	5.20, brs							
6″-OH	4.45, d (5.5)							
2‴-OH	4.66, d (6.0)							
3‴-OH	5.54, d (4.0)							
4‴-OH	5.13, brs							
2⁗-OH	5.02, m							
3⁗-OH	5.45, d (4.5)							
4⁗-OH	5.49, d (4.0)							
6⁗-OH	4.65, d (5.5)							
OH			4.93, brs					

^a 1^H NMR (500 MHz) and ^13^C NMR (125 MHz) measured in DMSO-*d*_6_. ^b 1^H NMR (400 MHz) and ^13^C NMR (100 MHz) measured in methanol-*d*_4_. ^c^ Overlapped with other signals. Assignments were supported with COSY, HMQC, and HMBC NMR spectra.

**Table 2:** Inhibition of Aβ peptide and NO production in HelaAPP and BV-2 microglial cells by the 14 isolated compounds (**1**–**14**).

Compounds	IC_50_ Value (µM) ^a^		
	Aβ42	NO	Cytotoxicity
**1**	-	>100	>100
**2**	>100	>100	>100
**3**	-	16.33 ± 2.94	>100
**4**	-	14.38 ± 8.68	>100
**5**	-	>100	>100
**6**	>100	>100	>100
**7**	-	>100	>100
**8**	-	>100	>100
**9**	-	>100	>100
**10**	-	>100	>100
**11**	-	>100	>100
**12**	>100	>100	>100
**13**	65.43 ± 6.71	>100	>100
**14**	-	17.44 ± 2.77	>100
Justicidine A ^b^	1.0 ± 0.043		
Dexamethasone ^b^		1.24 ± 0.104	>100

^a^ Cells were pretreated with isolated compounds (50 μM) for 1 h and incubated with 100 ng/mL LPS for 24 h. The results are presented as means ± SDs (n = 3). ^b^ Positive control—not detected.

## Data Availability

Data are contained within the article and Supplementary Materials.

## Supplementary Materials

The following supporting information can be downloaded at: https://www.mdpi.com/article/10.3390/plants13081094/s1. Figure S1. Inhibition of Aβ42 production from fractions of *E. hamiltonianus* in HelaAPP cells. Figure S2.1. HR-ESIMS spectrum of compound **1**. Figure S2.2. ^1^H-NMR spectrum of compound **1** in DMSO-*d*_6_ at 500 MHz. Figure S2.3. ^13^C-NMR spectrum of compound **1** in DMSO-*d*_6_ at 125 MHz. Figure S2.4. HSQC spectrum of compound **1** in DMSO-*d*_6_. Figure S2.5. COSY spectrum of compound **1** in DMSO-*d*_6._ Figure S2.6. HMBC spectrum of compound **1** in DMSO-*d*_6._ Figure S3.1. HR-ESIMS spectrum of compound **2**. Figure S3.2. ^1^H-NMR spectrum of compound **2** in DMSO-*d*_6_ at 500 MHz. Figure S3.3. ^13^C-NMR spectrum of compound **2** in DMSO-*d*_6_ at 125 MHz. Figure S3.4. HSQC spectrum of compound **2** in DMSO-*d*_6_. Figure S3.5. COSY spectrum of compound **2** in DMSO-*d*_6._ Figure S3.6. HMBC spectrum of compound **2** in DMSO-*d*_6_. Figure S3.7. LC-MS spectrum of compound **2**. Figure S3.8. Purity of compound **2**. Figure S3.9. LC-MS analysis of compound **2** compared to 30% extract. Figure S3.10. LC-MS analysis of compound **1** compared to 30% extract. Figure S4.1. HR-ESIMS spectrum of compound **10**. Figure S4.2. ^1^H-NMR spectrum of compound **10** in DMSO-*d*_6_ at 500 MHz. Figure S4.3. HSQC spectrum of compound **10** in DMSO-*d*_6._ Figure S4.4. COSY spectrum of compound **10** in DMSO-*d*_6._ Figure S4.5. HMBC spectrum of compound **10** in DMSO-*d*_6_. Figure S5.1. HR-ESIMS spectrum of compound 11. Figure S5.2. ^1^H-NMR spectrum of compound **11** in methanol-*d*_4_ at 400 MHz. Figure S5.3. ^13^C-NMR spectrum of compound **11** in methanol-*d*_4_ at 100 MHz. Figure S5.4. HSQC spectrum of compound **11** in methanol-*d*_4._ Figure S5.5. COSY spectrum of compound **11** in methanol-*d*_4._ Figure S5.6. HMBC spectrum of compound **11** in methanol-*d*_4_. Figure S6. HPLC pattern of sugar. Figure S7.1. ^1^H-NMR spectrum of compound **3** in methanol-*d*_4_ at 400 MHz. Figure S7.2. ^13^C-NMR spectrum of compound **3** in methanol-*d*_4_ at 100 MHz. Figure S8.1. ^1^H-NMR spectrum of compound **4** in DMSO-*d*_6_ at 400 MHz. Figure S8.2. ^13^C-NMR spectrum of compound **4** in DMSO-*d*_6_ at 100 MHz. Figure S9.1. ^1^H-NMR spectrum of compound **5** in DMSO-*d*_6_ at 400 MHz. Figure S9.2. ^13^C-NMR spectrum of compound **5** in DMSO-*d*_6_ at 100 MHz. Figure S10.1. ^1^H-NMR spectrum of compound **6** in DMSO-*d*_6_ at 400 MHz. Figure S10.2. ^13^C-NMR spectrum of compound **6** in DMSO-*d*_6_ at 100 MHz. Figure S11.1. ^1^H-NMR spectrum of compound **7** in DMSO-*d*_6_ at 400 MHz. Figure S11.2. ^13^C-NMR spectrum of compound **7** in DMSO-*d*_6_ at 100 MHz. Figure S12.1. ^1^H-NMR spectrum of compound **8** in DMSO-*d*_6_ at 400 MHz. Figure S12.2. ^13^C-NMR spectrum of compound **8** in DMSO-*d*_6_ at 100 MHz. Figure S13.1. ^1^H-NMR spectrum of compound **9** in DMSO-*d*_6_ at 400 MHz. Figure S13.2. ^13^C-NMR spectrum of compound **9** in DMSO-*d*_6_ at 100 MHz. Figure S14.1. ^1^H-NMR spectrum of compound **12** in DMSO-*d*_6_ at 400 MHz. Figure S14.2. ^13^C-NMR spectrum of compound **12** in DMSO-*d*_6_ at 100 MHz. Figure S15.1. ^1^H-NMR spectrum of compound **13** in DMSO-*d*_6_ at 400 MHz. Figure S15.2. ^13^C-NMR spectrum of compound **13** in DMSO-*d*_6_ at 100 MHz. Figure S16.1. ^1^H-NMR spectrum of compound **14** in DMSO-*d*_6_ at 400 MHz. Figure S16.2. ^13^C-NMR spectrum of compound **14** in DMSO at 100 MHz.
