# Supplementary material for: Neuroprotective Effects of Chemical Constituents of Leaves of Euonymus hamiltonianus Wall
Source: Plants (Basel). 2024 Apr 13;13(8):1094. doi: 10.3390/plants13081094 (PMC11054132; doi:10.3390/plants13081094)
Supplement: Supplementary file 1 [file plants-13-01094-s001.zip › plants-2937179-supplementary.pdf]

Supplementary Materials

Neuroprotective Effects of Chemical Constituents of Leaves of *Euonymus hamiltonianus* Wall.

Huynh Nguyen Khanh Tran [1,†], Jae Sik Yu [2,†], Tianqi Huang [1,2], Gakyung Lee [2], Hyo Sun Choi [1] and Hyun Ok Yang [2,*]

[1] Natural Products Research Center, Korea Institute of Science and Technology, Gangneung 25451, Republic of Korea; hnktran2404@gmail.com (H.N.K.T.); 618509@kist.re.kr (T.H.); ewchoi10@gmail.com (H.S.C.)
[2] Department of Integrative Biological Sciences and Industry & Convergence Research Center for Natural Products, Sejong University, 209 Neungdong-ro, Gwangjin-gu, Seoul 05006, Republic of Korea; jsyu@sejong.ac.kr (J.S.Y.); lgg1025@sejong.ac.kr (G.L.)
[*] Correspondence: hoyang@sejong.ac.kr; Tel.: +82-2-3408-1959; Fax: +82-2-3408-4336
[†] These authors contributed equally to this work.

List of Figures

Figure S1. Inhibition of Aβ42 production from fractions of *E. hamiltonianus* in HelaAPP cells.

Figure S2.1. HR-ESIMS spectrum of compound **1**

Figure S2.2. ^{1}H-NMR spectrum of compound **1** in DMSO-d_6 at 500 MHz

Figure S2.3. ^{13}C-NMR spectrum of compound **1** in DMSO-d_6 at 125 MHz

Figure S2.4. HSQC spectrum of compound **1** in DMSO-d_6

Figure S2.5. COSY spectrum of compound **1** in DMSO-d_6

Figure S2.6. HMBC spectrum of compound **1** in DMSO-d_6

Figure S3.1. HR-ESIMS spectrum of compound **2**

Figure S3.2. ^{1}H-NMR spectrum of compound **2** in DMSO-d_6 at 500 MHz

Figure S3.3. ^{13}C-NMR spectrum of compound **2** in DMSO-d_6 at 125 MHz

Figure S3.4. HSQC spectrum of compound **2** in DMSO-d_6

Figure S3.5. COSY spectrum of compound **2** in DMSO-d_6

Figure S3.6. HMBC spectrum of compound **2** in DMSO-d_6

Figure S3.7. LC-MS spectrum of compound **2**

Figure S3.8. Purity of compound **2**

Figure S3.9. LC-MS analysis of compound **2** compared to 30% extract

Figure S3.10. LC-MS analysis of compound **1** compared to 30% extract

Figure S4.1. HR-ESIMS spectrum of compound **10**

Figure S4.2. ^{1}H-NMR spectrum of compound **10** in DMSO-d_6 at 500 MHz

Figure S4.3. HSQC spectrum of compound **10** in DMSO-d_6

Figure S4.4. COSY spectrum of compound **10** in DMSO-d_6

Figure S4.5. HMBC spectrum of compound **10** in DMSO-d_6

Figure S5.1. HR-ESIMS spectrum of compound **11**

Figure S5.2. ^{1}H-NMR spectrum of compound **11** in methanol-d_4 at 400 MHz

Figure S5.3. ^{13}C-NMR spectrum of compound **11** in methanol-d_4 at 100 MHz

Figure S5.4. HSQC spectrum of compound **11** in methanol-d_4

Figure S5.5. COSY spectrum of compound **11** in methanol-d_4

Figure S5.6. HMBC spectrum of compound **11** in methanol-d_4

Figure S6. HPLC pattern of sugar

Figure S7.1. ^{1}H-NMR spectrum of compound **3** in methanol-d_4 at 400 MHz

Figure S7.2. ^{13}C-NMR spectrum of compound **3** in methanol-d_4 at 100 MHz

Figure S8.1. ^{1}H-NMR spectrum of compound **4** in DMSO-d_6 at 400 MHz

Figure S8.2. ^{13}C-NMR spectrum of compound **4** in DMSO-d_6 at 100 MHz

Figure S9.1. ^{1}H-NMR spectrum of compound **5** in DMSO-d_6 at 400 MHz

Figure S9.2. ^{13}C-NMR spectrum of compound **5** in DMSO-d_6 at 100 MHz

Figure S10.1. ^{1}H-NMR spectrum of compound **6** in DMSO-d_6 at 400 MHz

Figure S10.2. ^{13}C-NMR spectrum of compound **6** in DMSO-d_6 at 100 MHz

Figure S11.1. ^{1}H-NMR spectrum of compound **7** in DMSO-d_6 at 400 MHz

Figure S11.2. ^{13}C-NMR spectrum of compound **7** in DMSO-d_6 at 100 MHz

Figure S12.1. ^{1}H-NMR spectrum of compound **8** in DMSO-d_6 at 400 MHz

Figure S12.2. ^{13}C-NMR spectrum of compound **8** in DMSO-d_6 at 100 MHz

Figure S13.1. ^{1}H-NMR spectrum of compound **9** in DMSO-d_6 at 400 MHz

Figure S13.2. ^{13}C-NMR spectrum of compound **9** in DMSO-d_6 at 100 MHz

Figure S14.1. ^{1}H-NMR spectrum of compound **12** in DMSO-d_6 at 400 MHz

Figure S14.2. ^{13}C-NMR spectrum of compound **12** in DMSO-d_6 at 100 MHz

Figure S15.1. ^{1}H-NMR spectrum of compound **13** in DMSO-d_6 at 400 MHz

Figure S15.2. ^{13}C-NMR spectrum of compound **13** in DMSO-d_6 at 100 MHz

Figure S16.1. ^{1}H-NMR spectrum of compound **14** in DMSO-d_6 at 400 MHz

Figure S16.2. ^{13}C-NMR spectrum of compound **14** in DMSO at 100 MHz

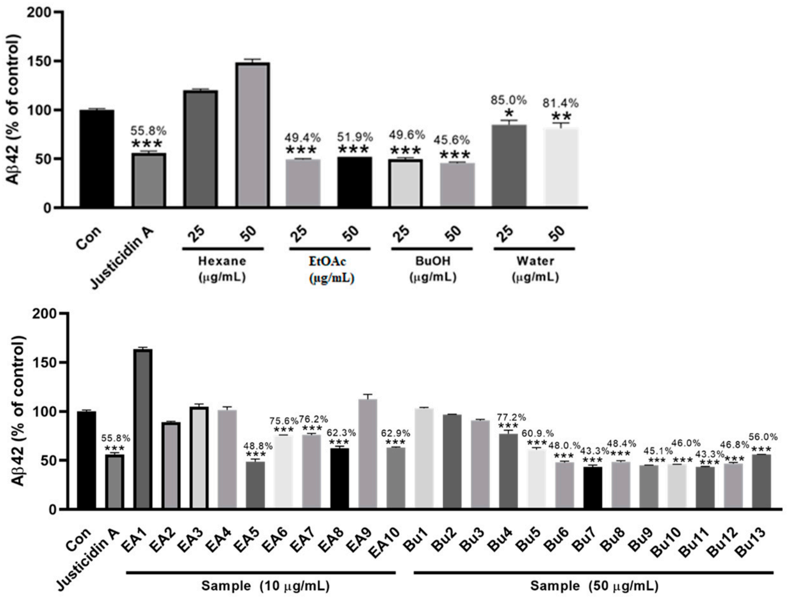

Figure S1. Inhibition of Aβ42 production from fractions of *E. hamiltonianus* in HelaAPP cells.

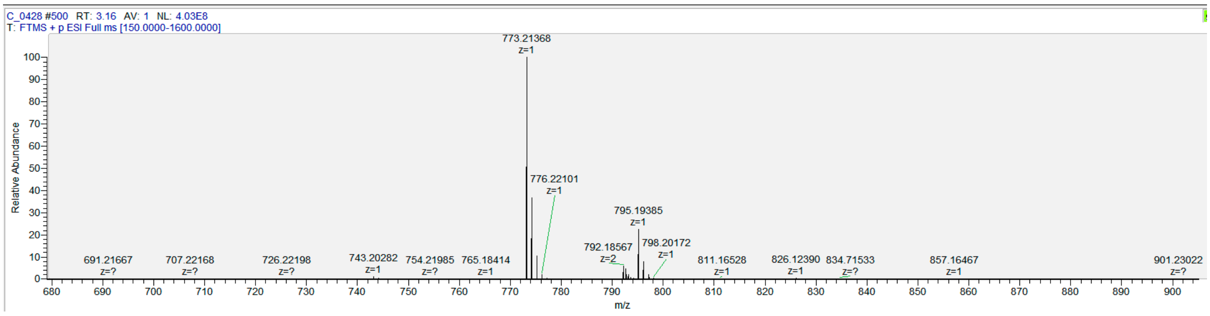

Elemental composition search on mass 773.21

m/z= 768.21-778.21

m/z	Theo. Mass	Delta (ppm)	RDB equiv.	Composition
773.2138	773.2135	0.42	13.5	$C_{33}H_{41}O_{21}$
	773.2076	8.02	22.5	$C_{40}H_{37}O_{16}$
	773.2287	-19.31	17.5	$C_{37}H_{41}O_{18}$
	773.1982	20.15	9.5	$C_{29}H_{41}O_{24}$
	773.2346	-26.91	8.5	$C_{30}H_{45}O_{23}$
	773.1924	27.75	18.5	$C_{36}H_{37}O_{19}$
	773.2499	-46.64	12.5	$C_{34}H_{45}O_{20}$
	773.1771	47.48	14.5	$C_{32}H_{37}O_{22}$
	773.2557	-54.23	3.5	$C_{27}H_{49}O_{25}$
	773.1712	55.07	23.5	$C_{39}H_{33}O_{17}$

Figure S2.1. HR-ESIMS spectrum of compound **1**

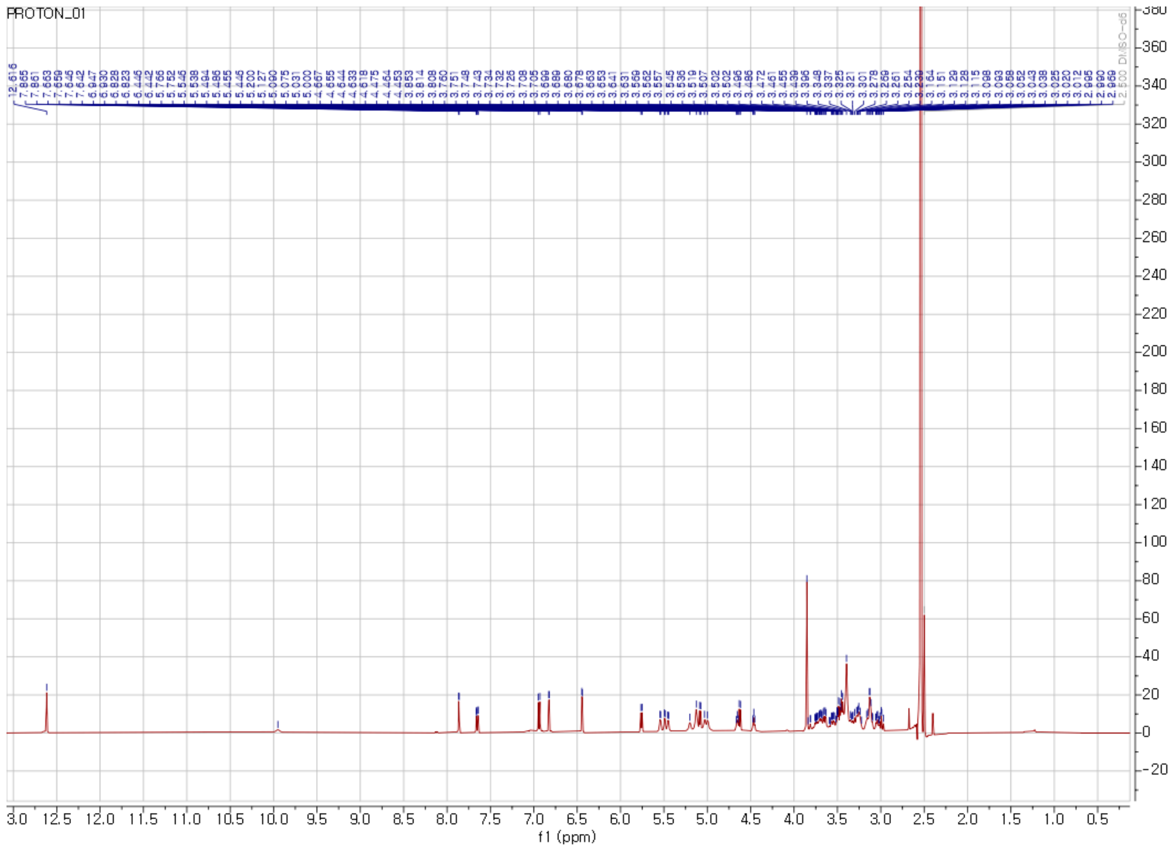

Figure S2.2. ^{1}H-NMR spectrum of compound **1** in DMSO-d_6 at 500 MHz

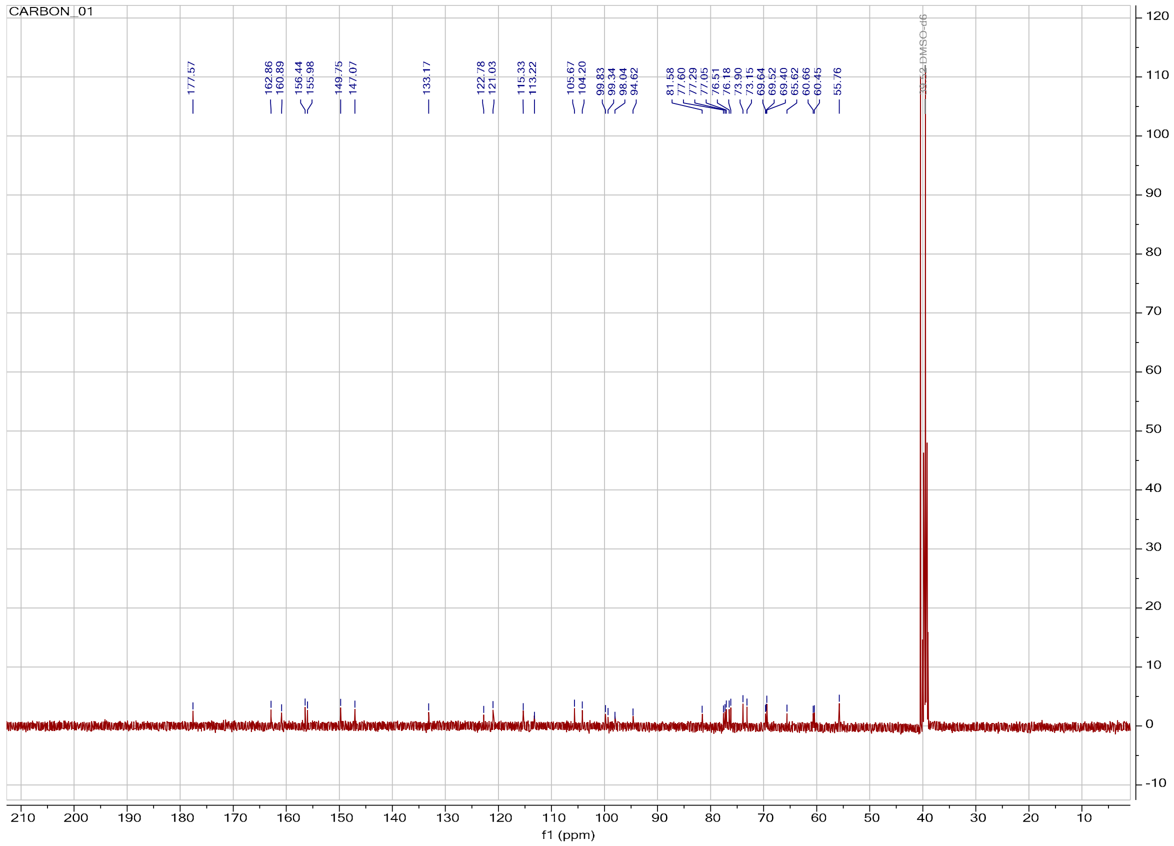

Figure S2.3. ^{13}C-NMR spectrum of compound **1** in DMSO-d_6 at 125 MHz

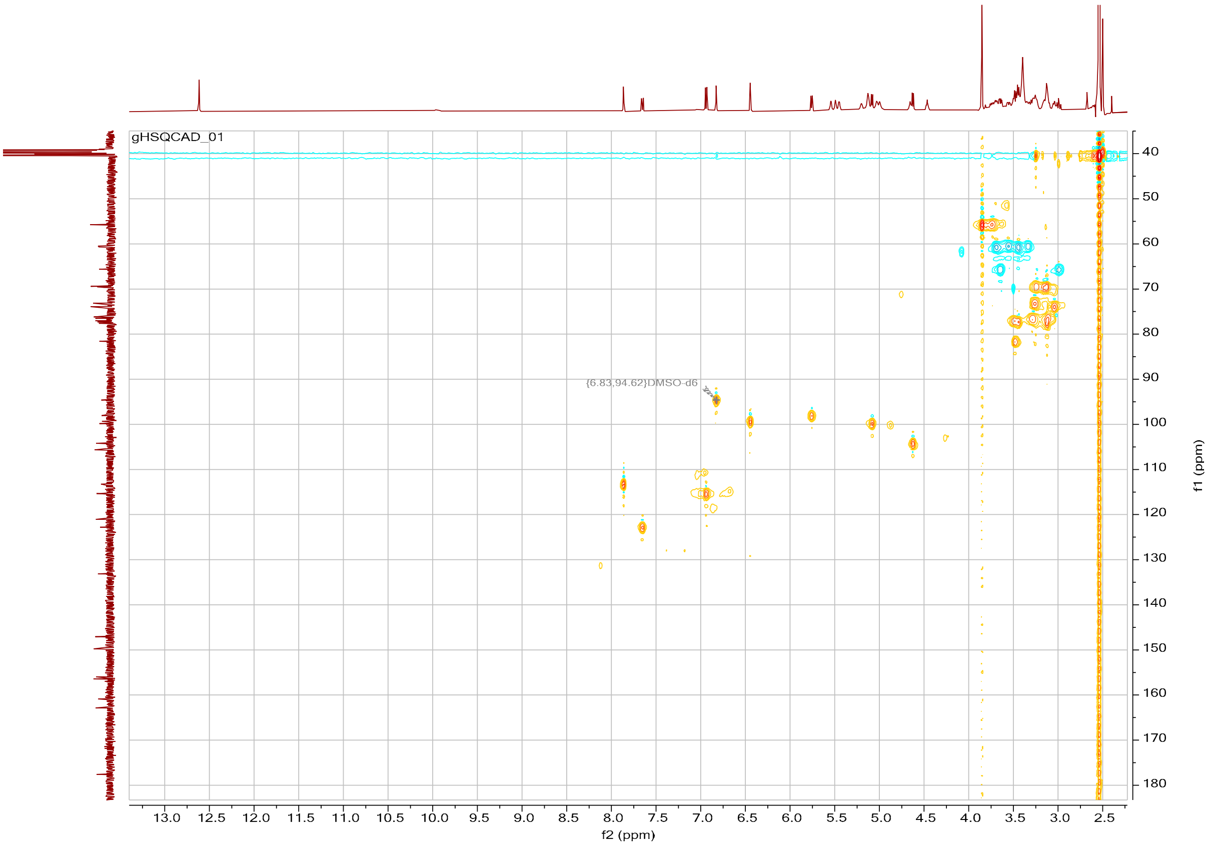

Figure S2.4. HSQC spectrum of compound **1** in DMSO-d_6

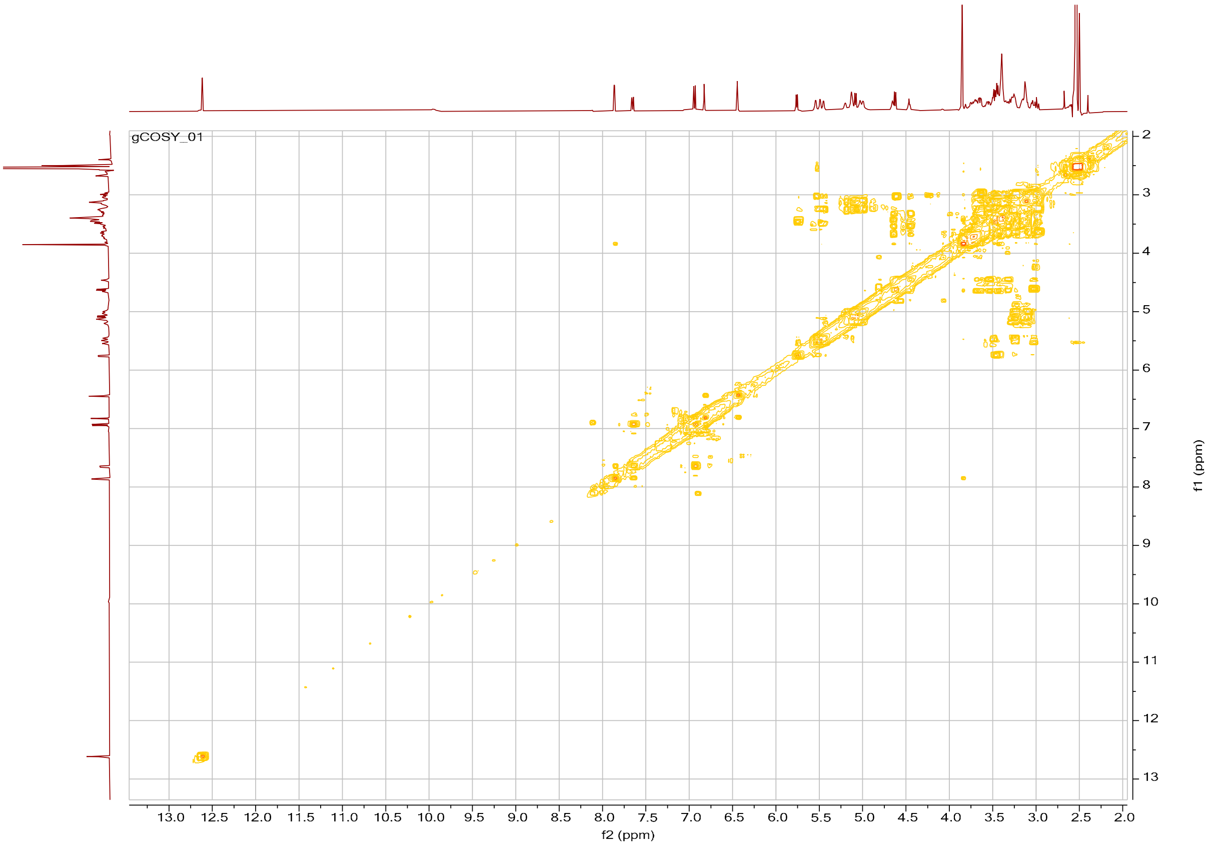

Figure S2.5. COSY spectrum of compound **1** in DMSO-d_6

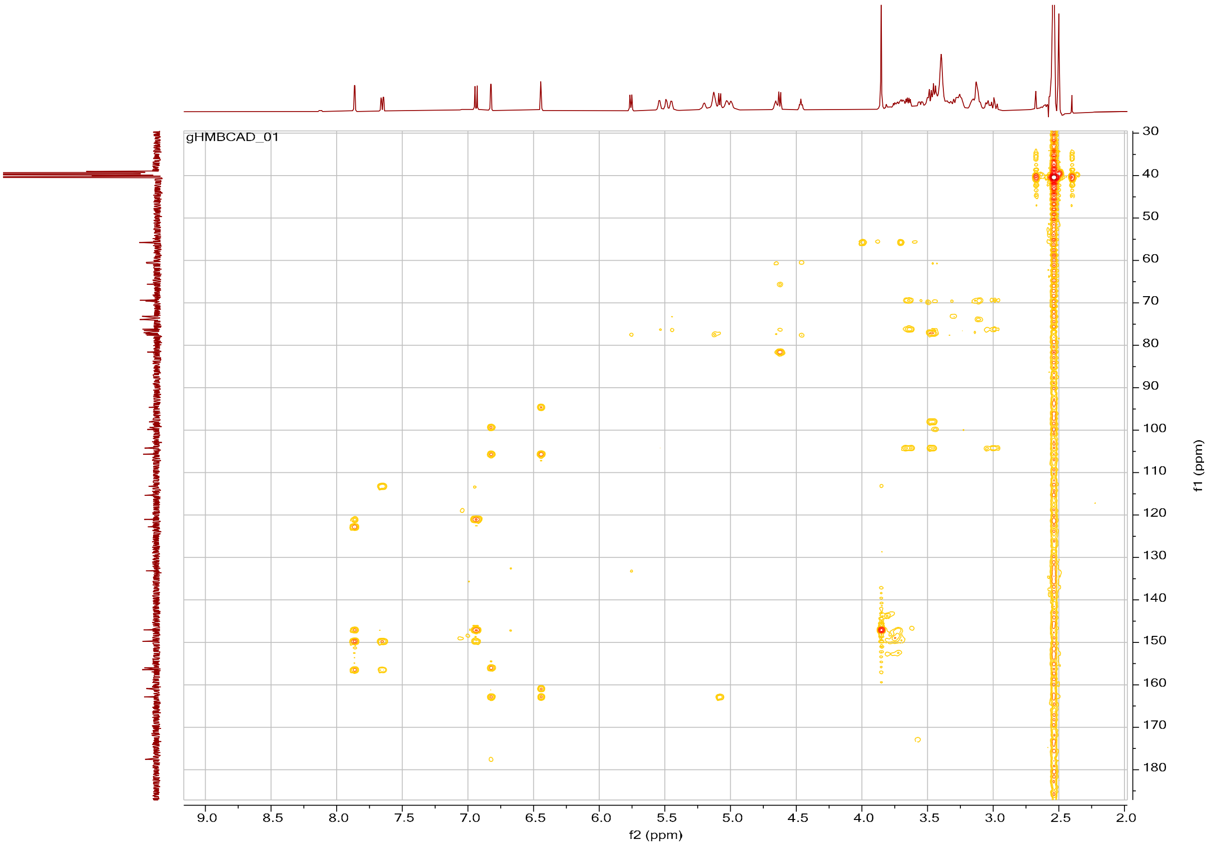

Figure S2.6. HMBC spectrum of compound **1** in DMSO-d_6

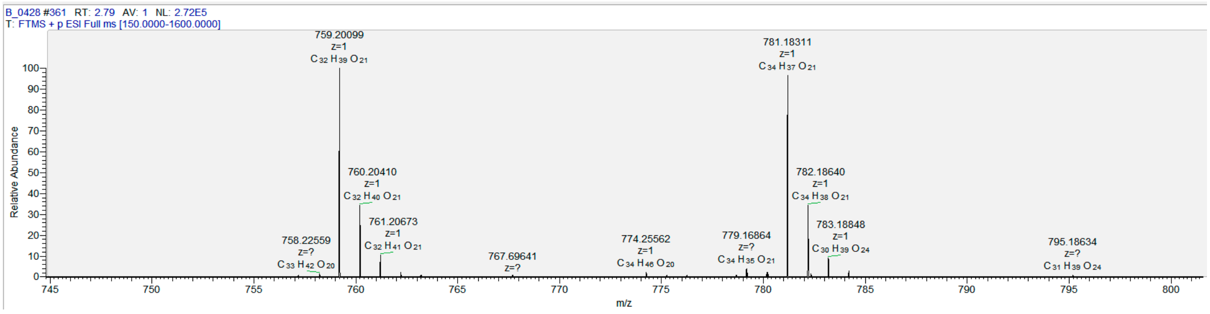

Elemental composition search on mass 759.20

m/z= 754.20-764.20

m/z	Theo. Mass	Delta (ppm)	RDB equiv.	Composition
759.2010	759.1978	4.16	13.5	$C_{32}H_{39}O_{21}$
	759.2190	-23.67	8.5	$C_{29}H_{43}O_{23}$
	759.1826	24.25	9.5	$C_{28}H_{39}O_{24}$
	759.1767	31.99	18.5	$C_{35}H_{35}O_{19}$
	759.2342	-43.77	12.5	$C_{33}H_{43}O_{20}$
	759.2401	-51.51	3.5	$C_{26}H_{47}O_{25}$
	759.1614	52.08	14.5	$C_{31}H_{35}O_{22}$
	759.2553	-71.60	7.5	$C_{30}H_{47}O_{22}$
	759.1462	72.18	10.5	$C_{27}H_{35}O_{25}$
	759.1403	79.91	19.5	$C_{34}H_{31}O_{20}$

Figure S3.1. HR-ESIMS spectrum of compound **2**

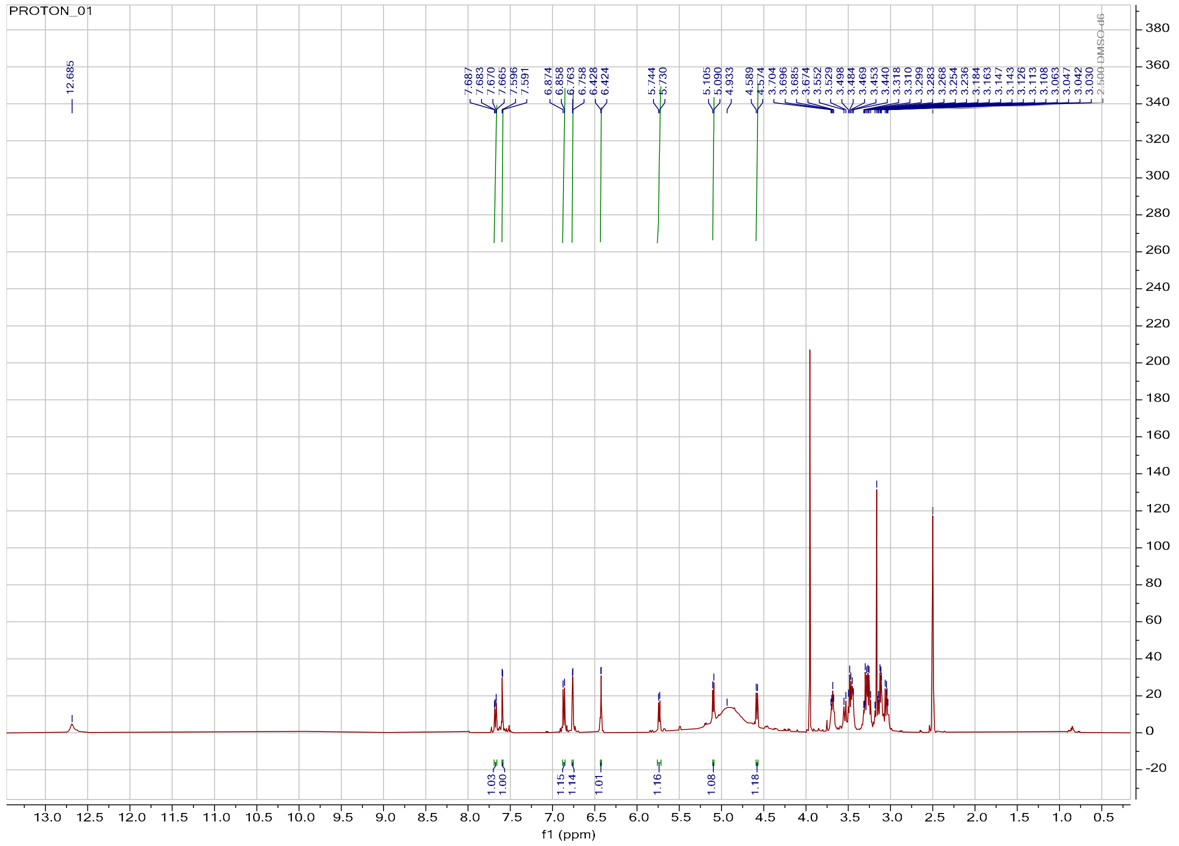

Figure S3.2. ^{1}H-NMR spectrum of compound **2** in DMSO-d_6 at 500 MHz

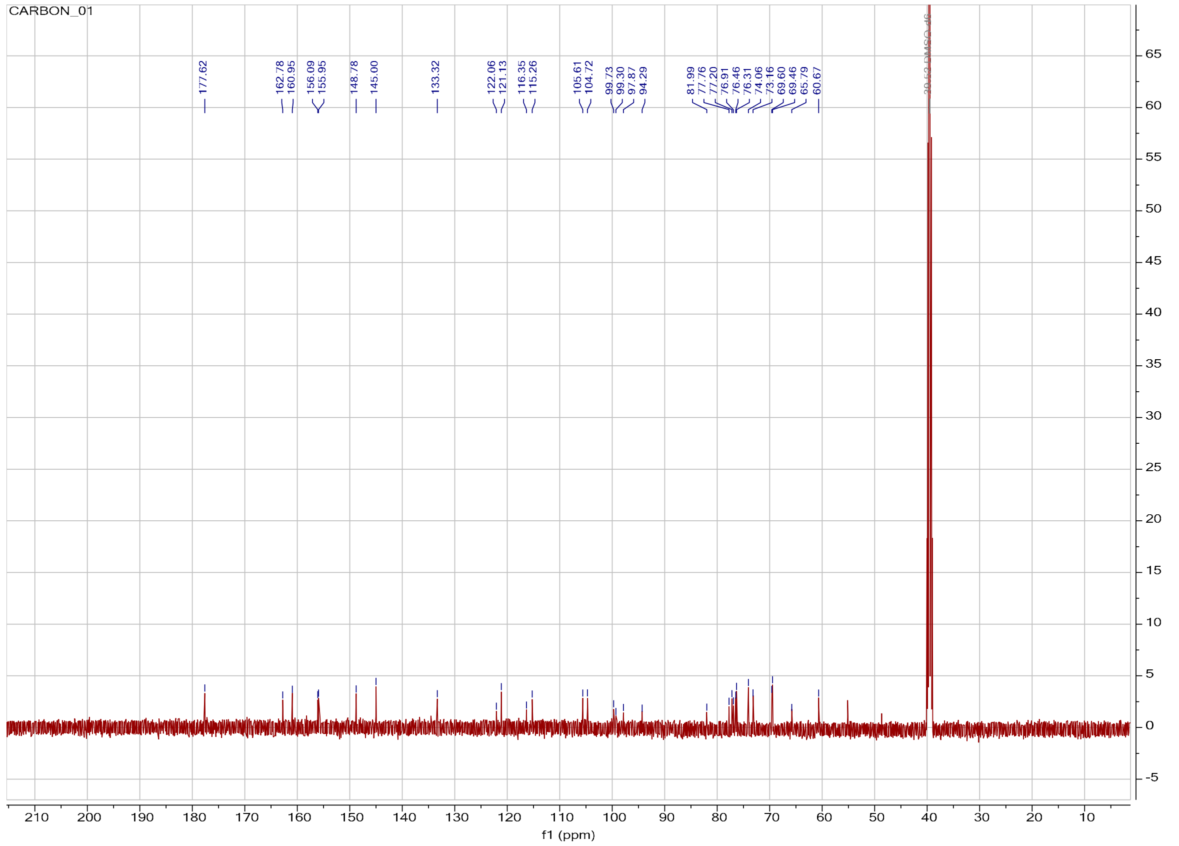

Figure S3.3. ^{13}C-NMR spectrum of compound **2** in DMSO-d_6 at 125 MHz

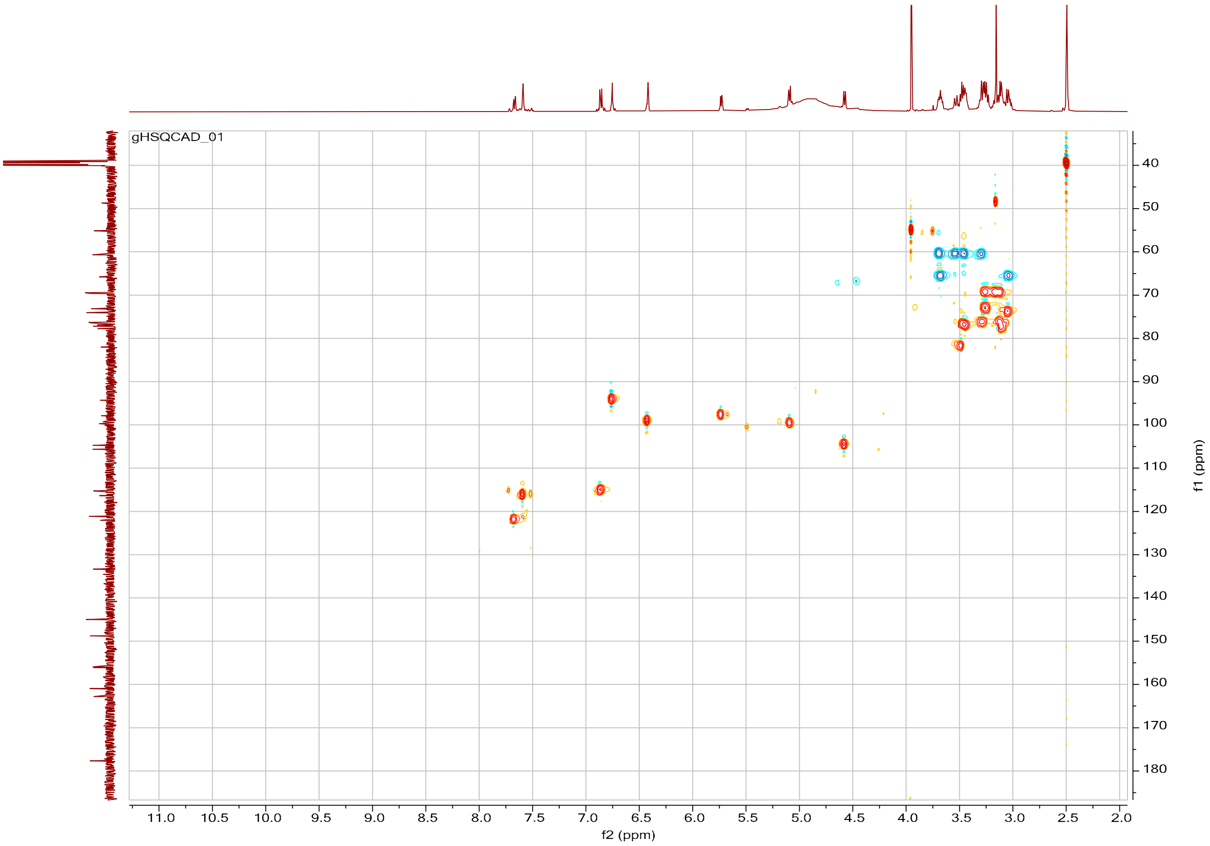

Figure S3.4. HSQC spectrum of compound **2** in DMSO-d_6

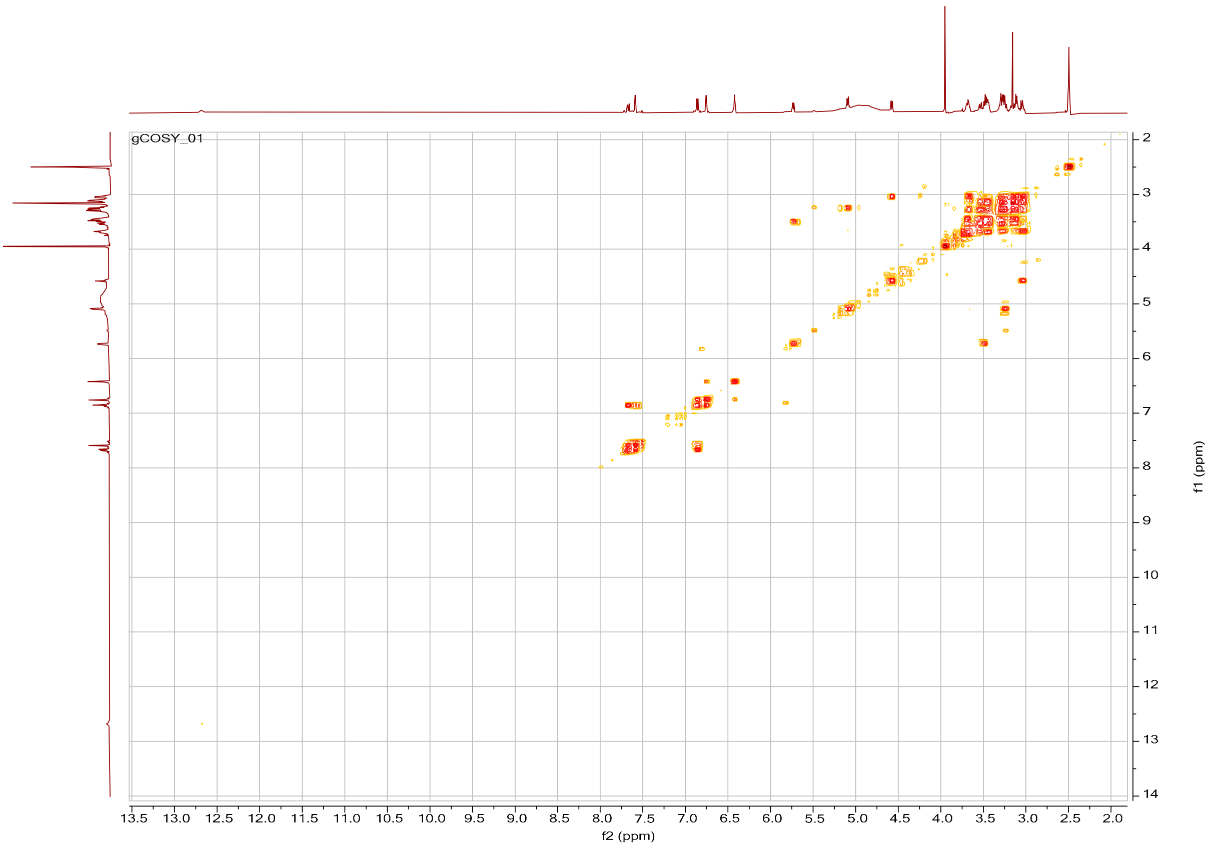

Figure S3.5. COSY spectrum of compound **2** in DMSO-d_6

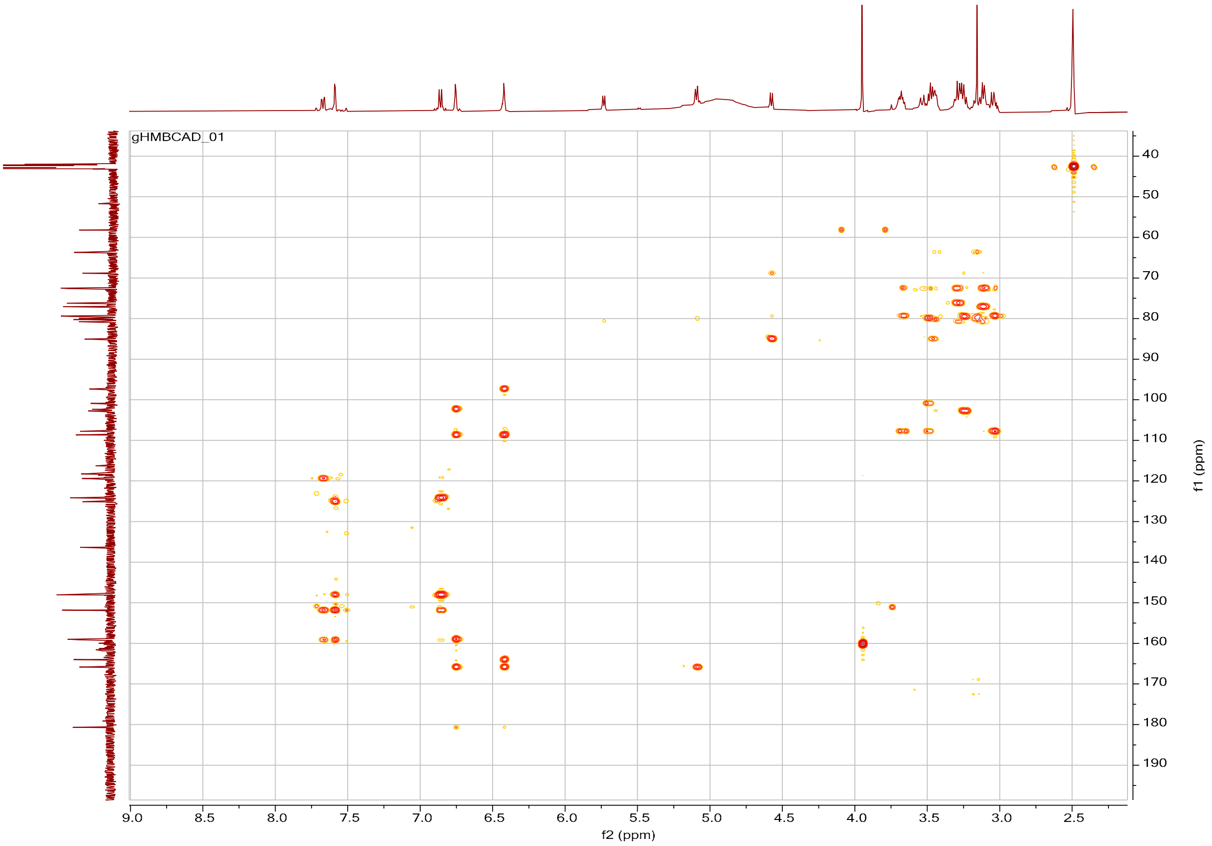

Figure S3.6. HMBC spectrum of compound **2** in DMSO-d_6

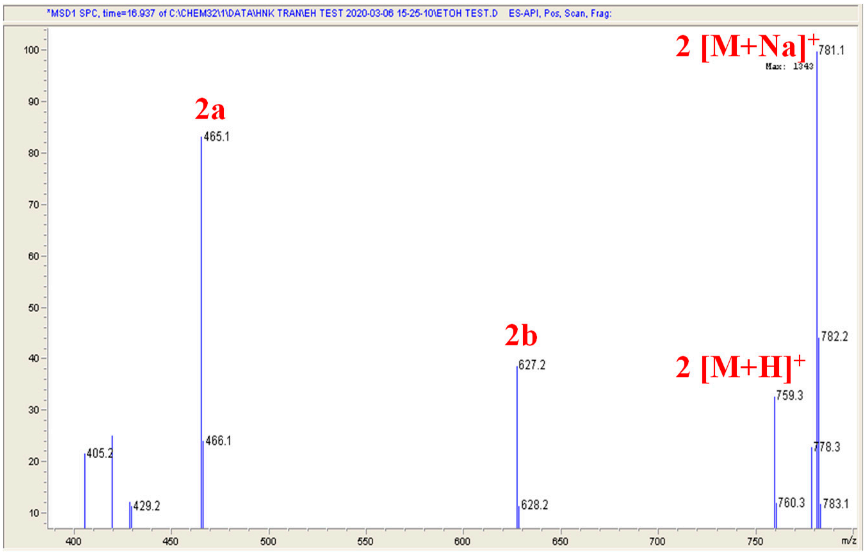

Figure S3.7. LC-MS chromatogram of compound **2**

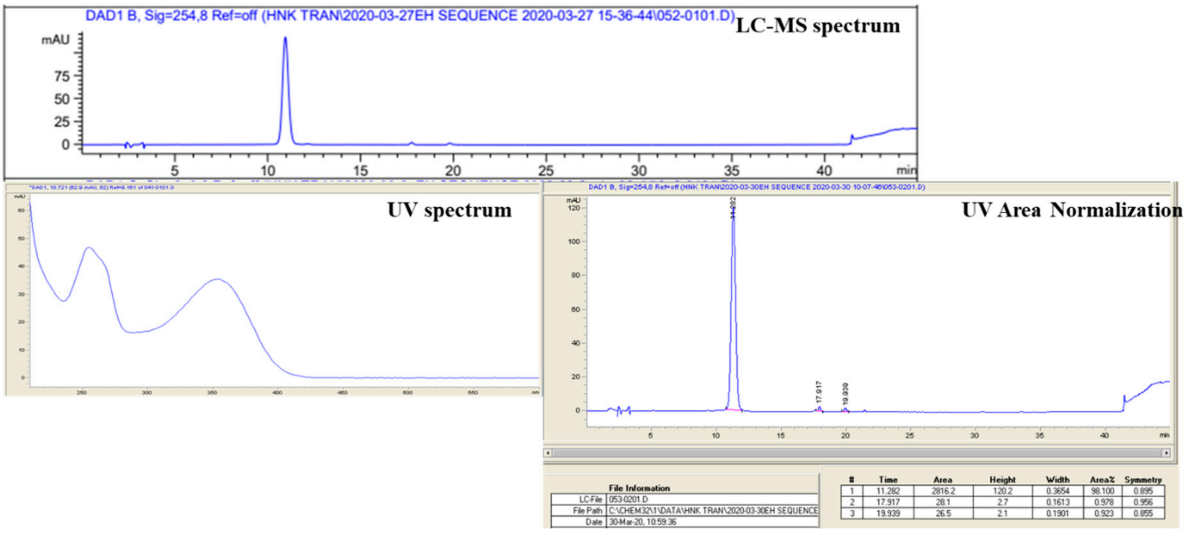

Figure S3.8 Purity of Compound **2**

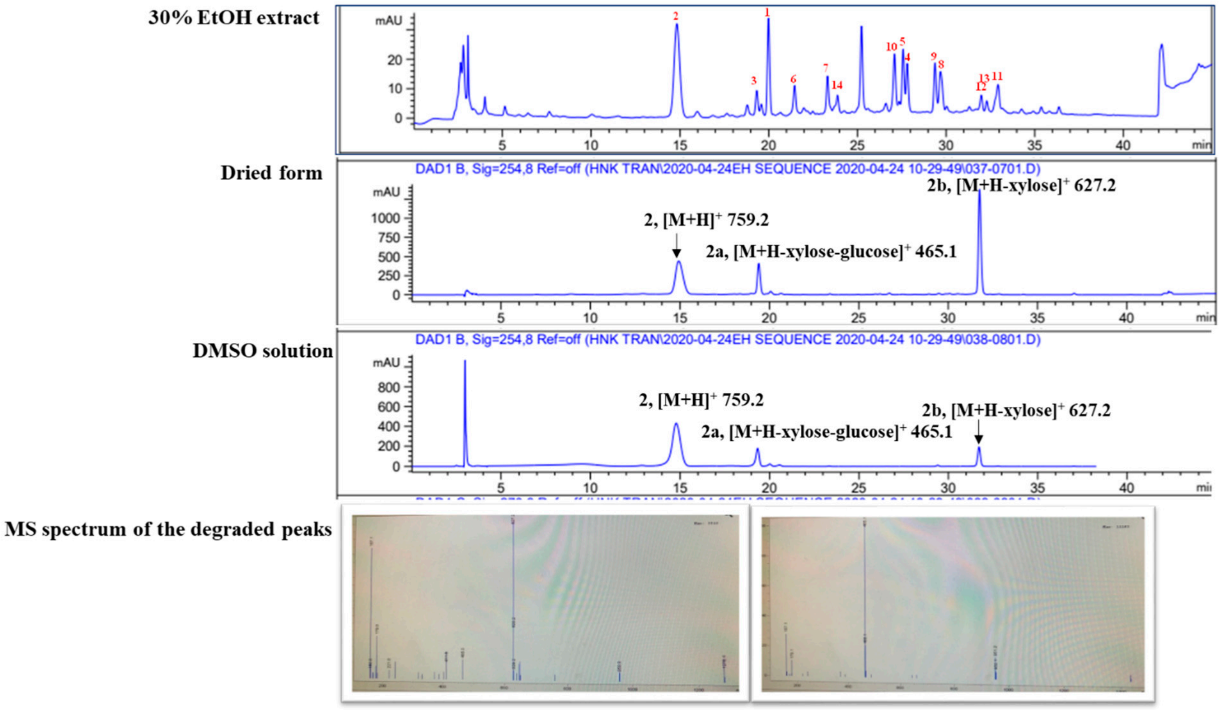

Figure S3.9. LC-MS analysis of compound **2** compared to 30% extract

30% EtOH extract

Compound 1

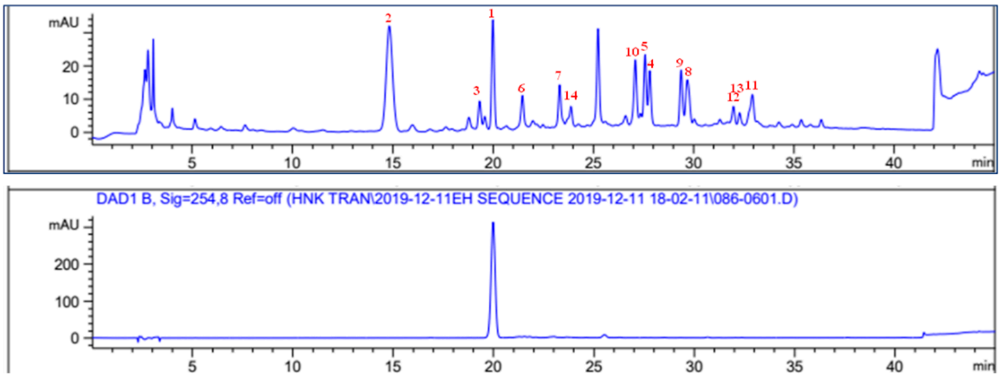

Figure S3.10. LC-MS analysis of compound **1** compared to 30% extract

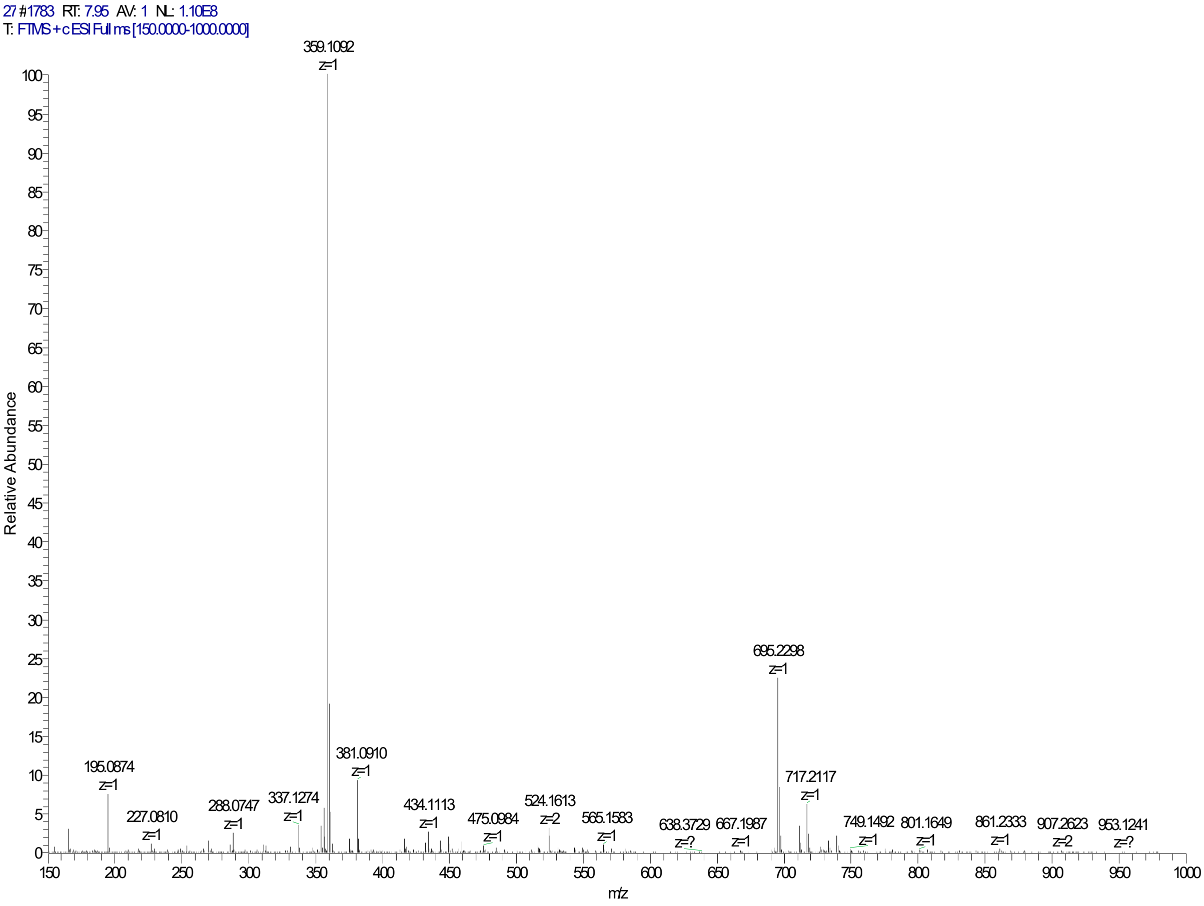

Elemental composition search on mass 359.11

m/z= 354.11-364.11

m/z	Theo. Mass	Delta (ppm)	RDB equiv.	Composition
359.1092	359.1101	-2.60	7.5	$C_{17}H_{20}O_7Na$
	359.1077	4.10	4.5	$C_{15}H_{21}O_7Na_2$
	359.1067	7.06	19.5	$C_{26}H_{15}O_2$
	359.1125	-9.30	10.5	$C_{19}H_{19}O_7$
	359.1053	10.79	1.5	$C_{13}H_{22}O_7Na_3$
	359.1043	13.75	16.5	$C_{24}H_{16}O_2Na$
	359.1158	-18.29	-0.5	$C_{13}H_{24}O_4Na_5$
	359.1018	20.45	13.5	$C_{22}H_{17}O_2Na_2$
	359.1182	-24.99	2.5	$C_{15}H_{23}O_4Na_4$
	359.1184	-25.65	1.5	$C_{12}H_{23}O_{12}$

Figure S4.1. HR-ESIMS spectrum of compound **10**

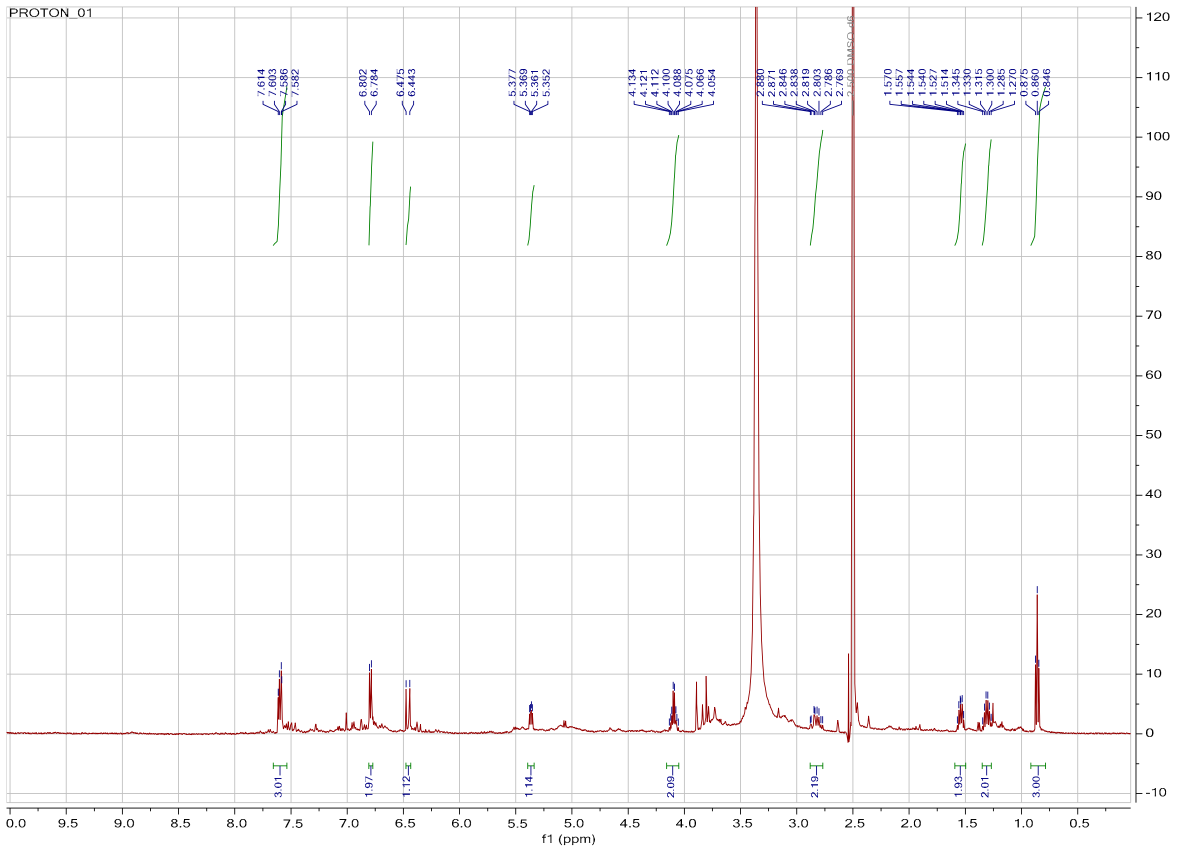

Figure S4.2. ^{1}H-NMR spectrum of compound **10** in DMSO-d_6 at 500 MHz

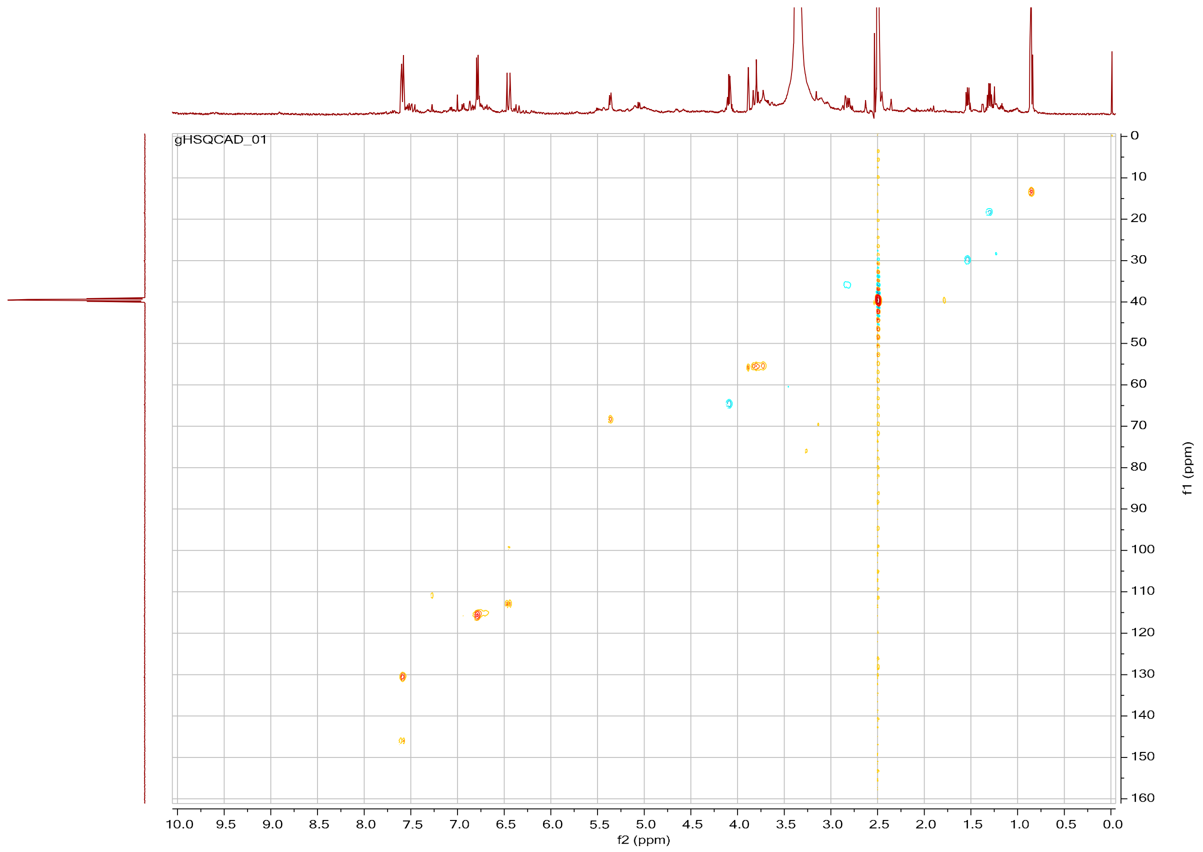

Figure S4.3. HSQC spectrum of compound **10** in DMSO-d_6

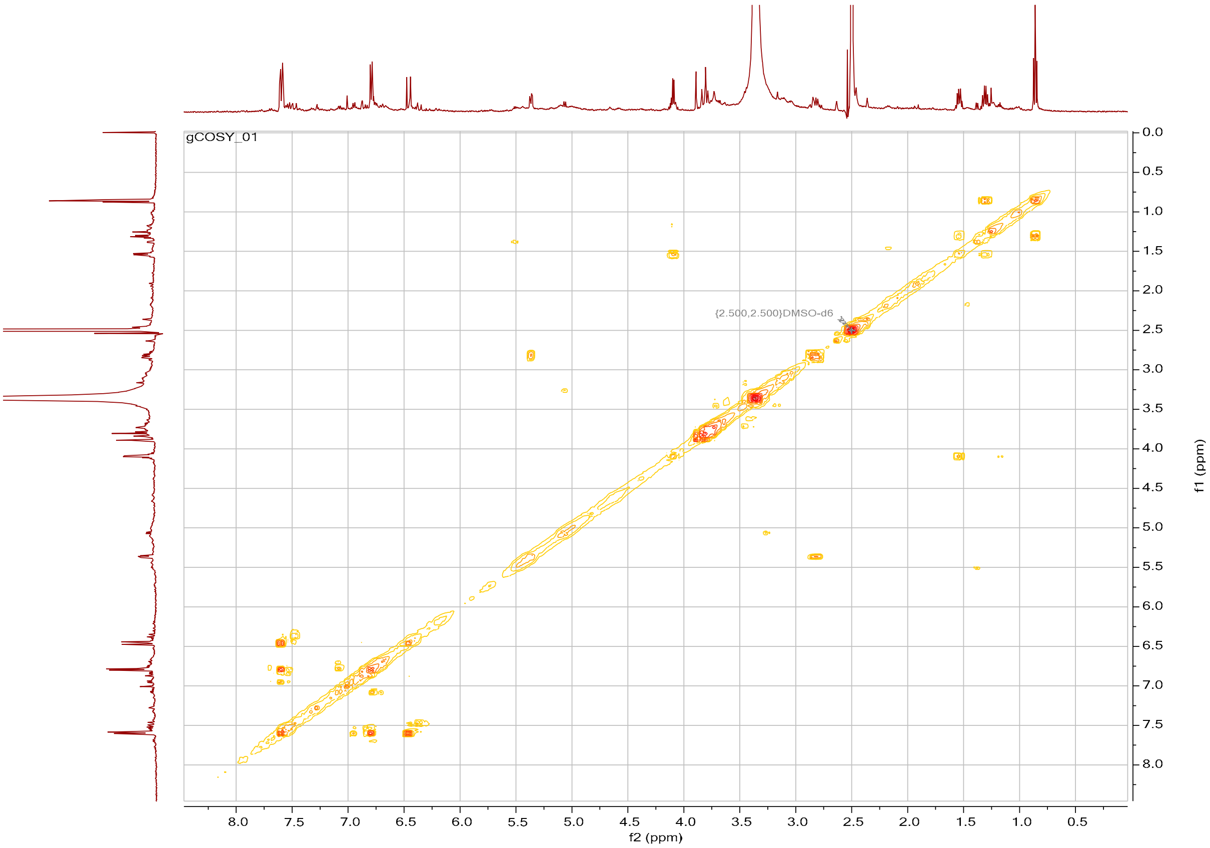

Figure S4.4. COSY spectrum of compound **10** in DMSO-d_6

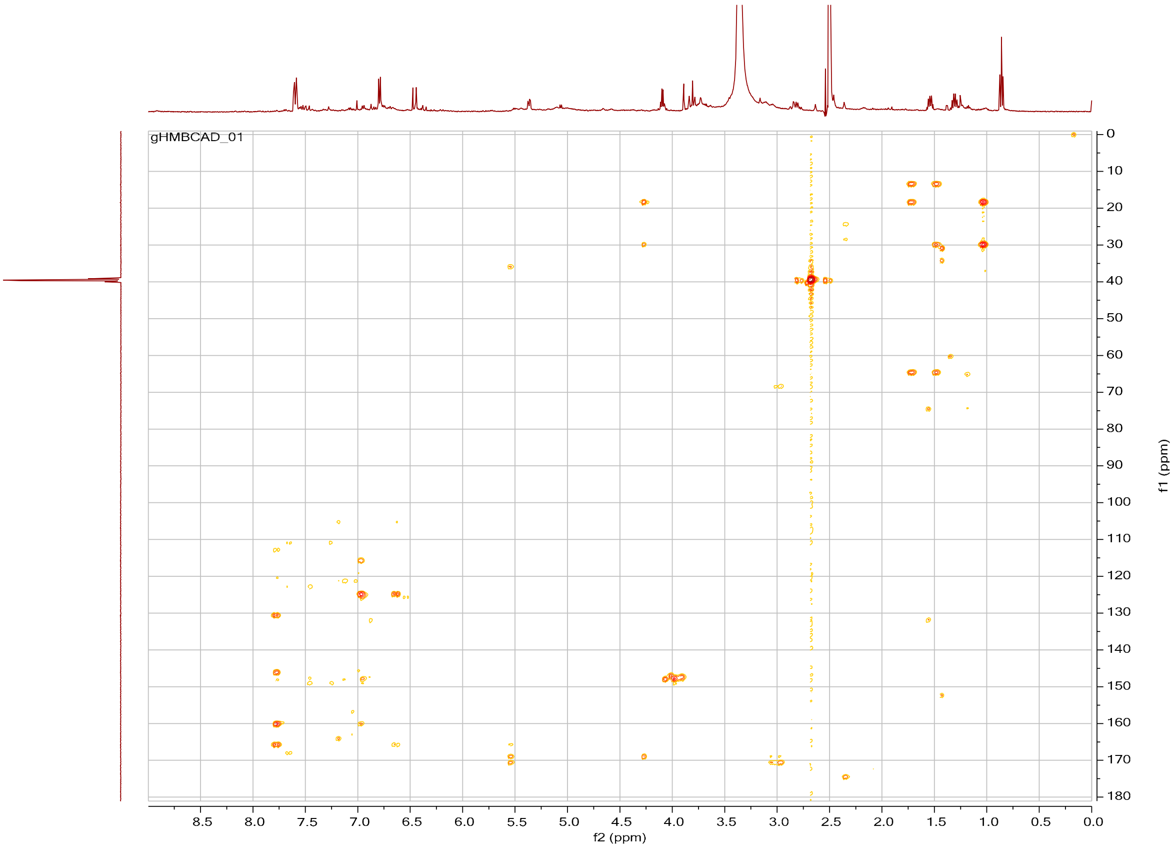

Figure S4.5. HMBC spectrum of compound **10** in DMSO-d_6

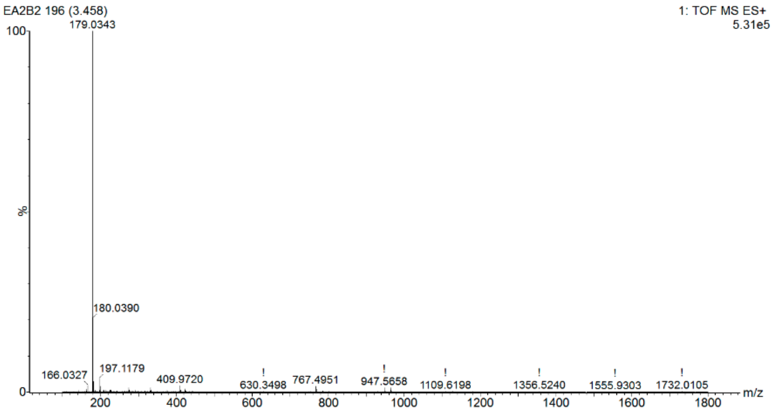

Elemental Composition Report Page 1

Single Mass Analysis
Tolerance = 5.0 mDa / DBE: min = -1.5, max = 50.0
Element prediction: Off
Number of isotope peaks used for i-FIT = 3

Monoisotopic Mass, Even Electron Ions
27 formula(e) evaluated with 1 results within limits (all results (up to 1000) for each mass)
Elements Used:
C: 0-500 H: 0-1000 O: 0-20
EA2B2 196 (3.458)
1: TOF MS ES+

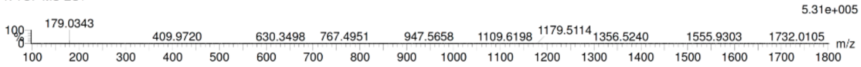

Minimum:				-1.5				
Maximum:		5.0	10.0	50.0				
Mass	Calc. Mass	mDa	PPM	DBE	i-FIT	Norm	Conf(%)	Formula
179.0343	179.0344	-0.1	-0.6	6.5	754.8	n/a	n/a	C9 H7 O4

Figure S5.1. HR-ESIMS spectrum of compound **11**

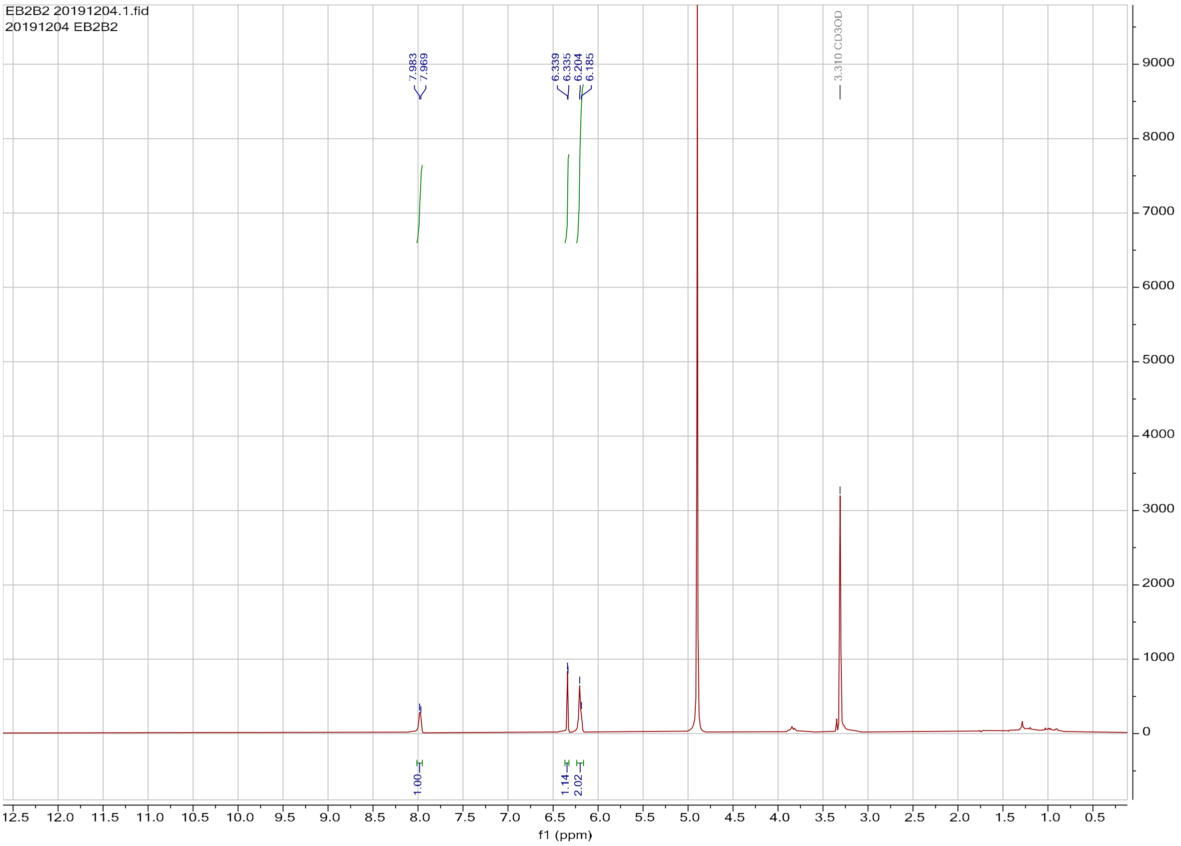

Figure S5.2. ^{1}H-NMR spectrum of compound **11** in methanol-*d*4 at 400 MHz

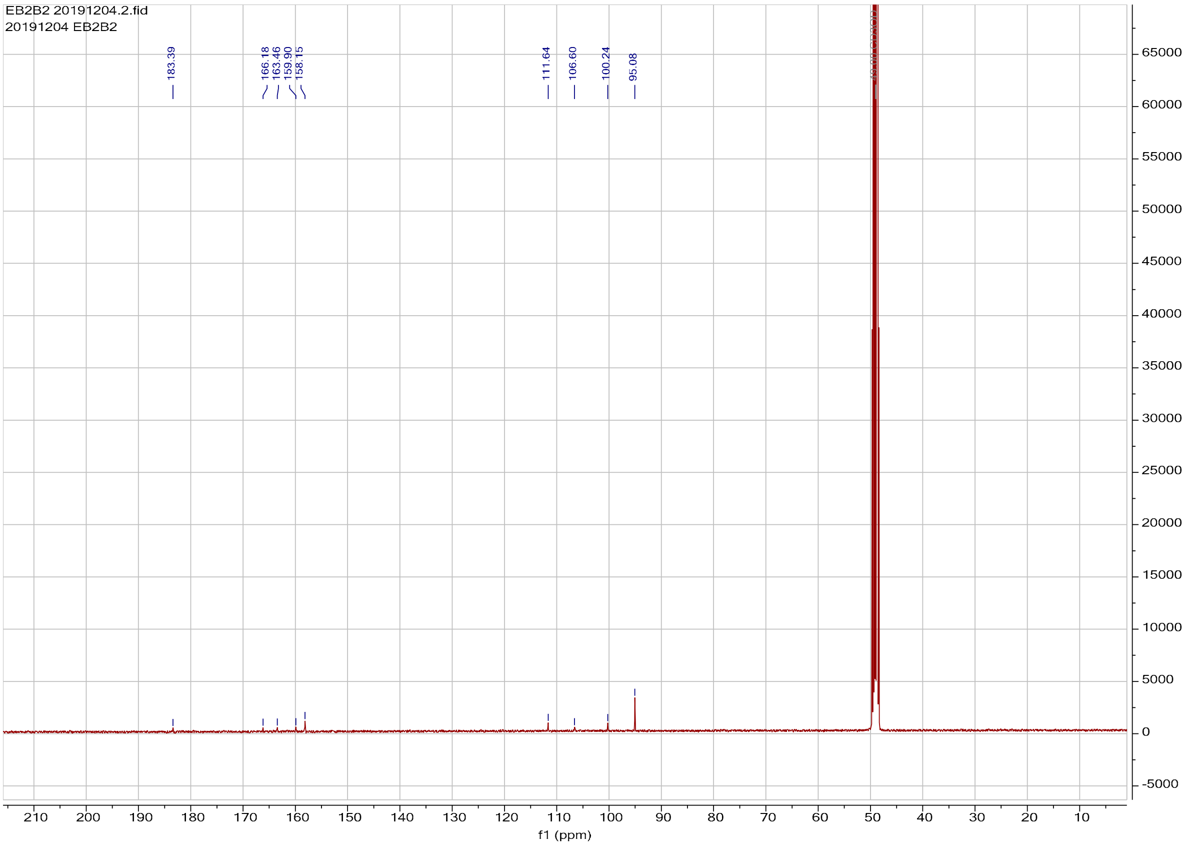

Figure S5.3. ^{13}C-NMR spectrum of compound **11** in methanol-*d*4 at 100 MHz

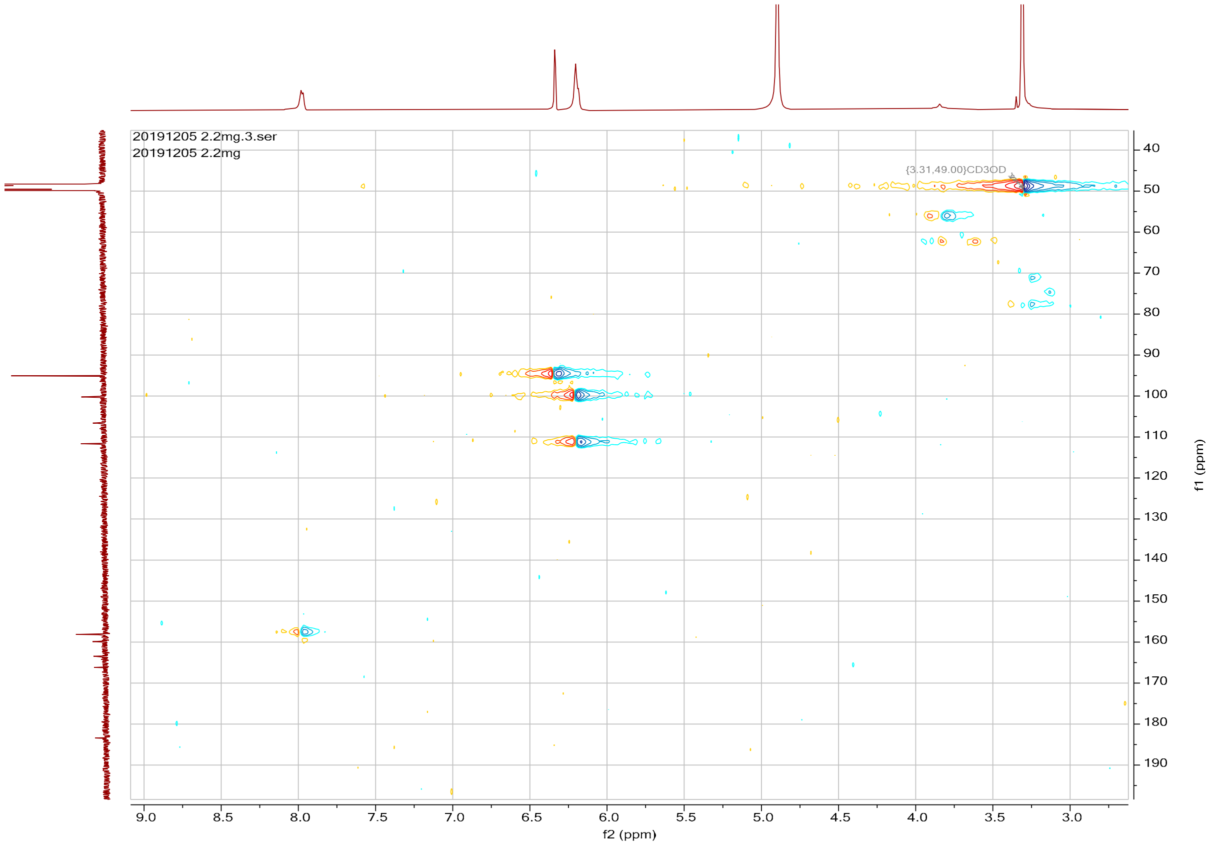

Figure S5.4. HSQC spectrum of compound **11** in methanol-*d*4

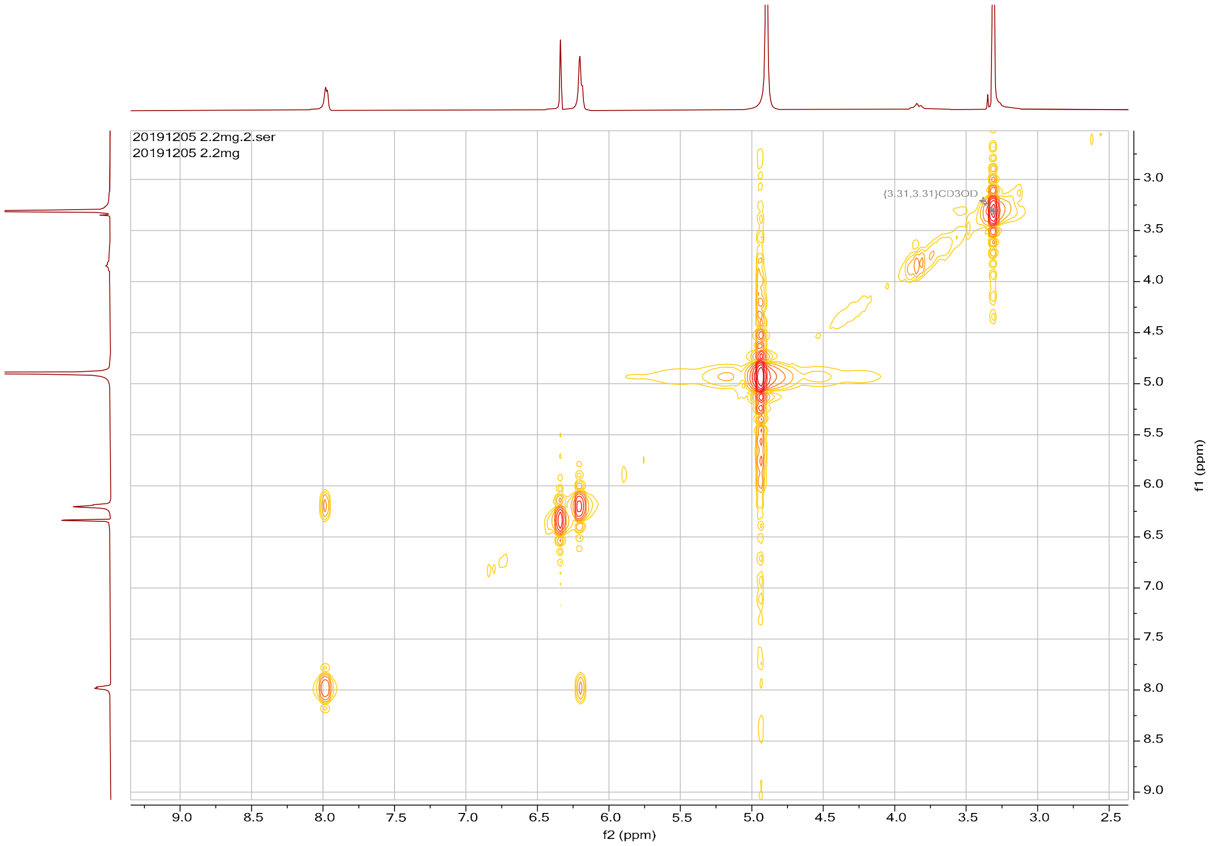

Figure S5.5. COSY spectrum of compound **11** in methanol-*d*4

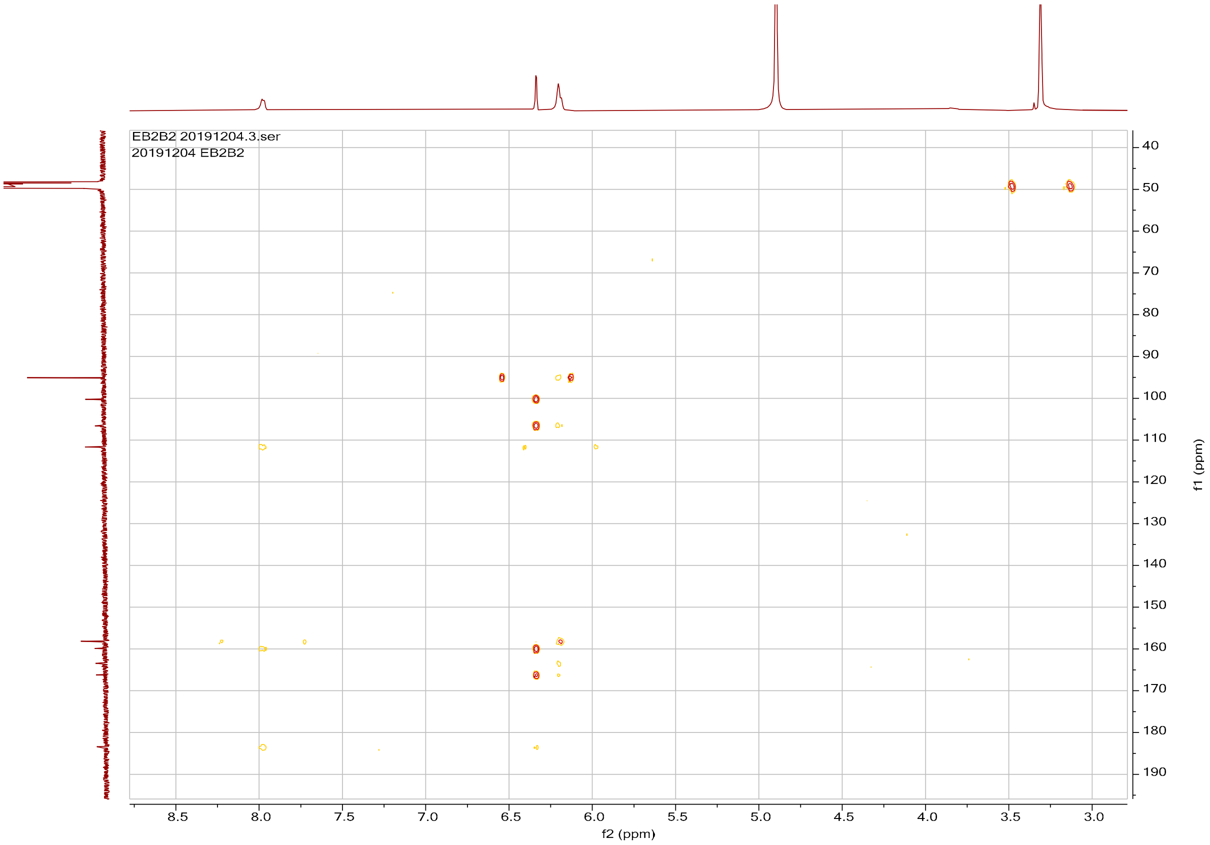

Figure S5.6. HMBC spectrum of compound **11** in methanol-*d*4

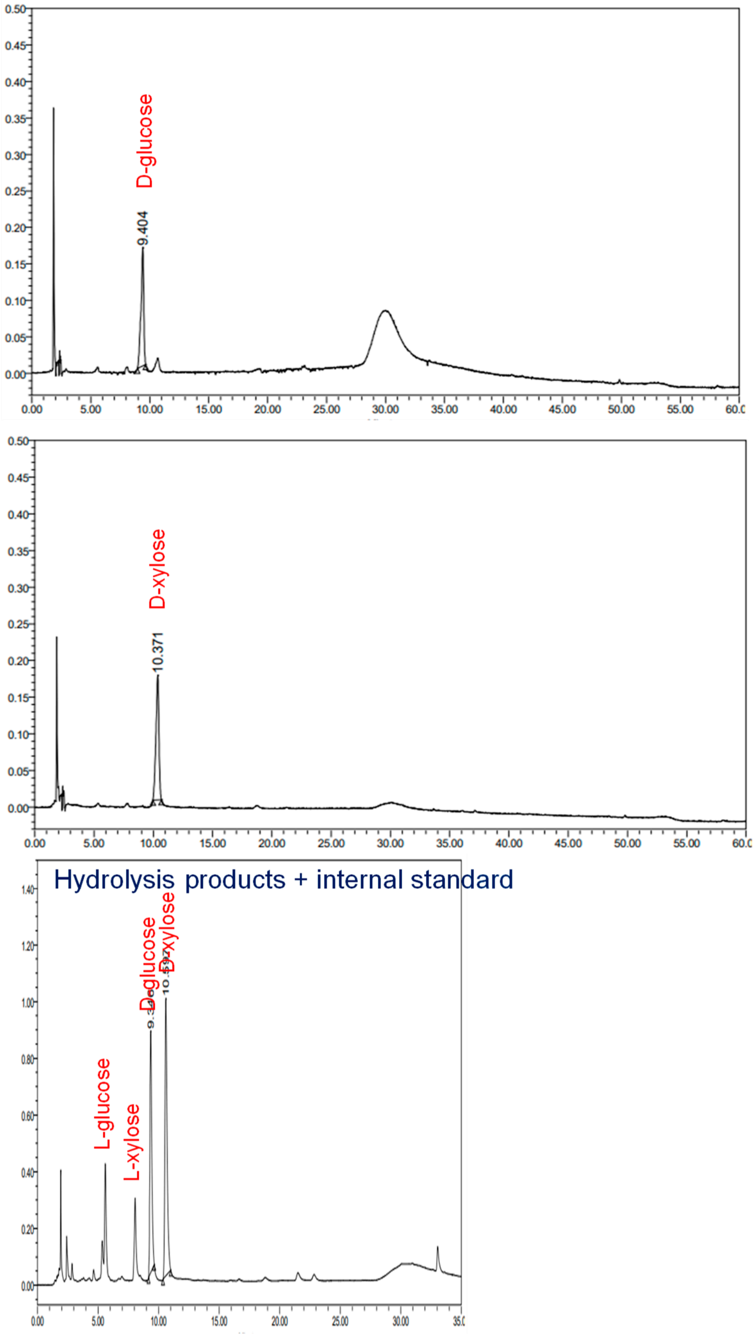

Figure S6. HPLC pattern of sugar

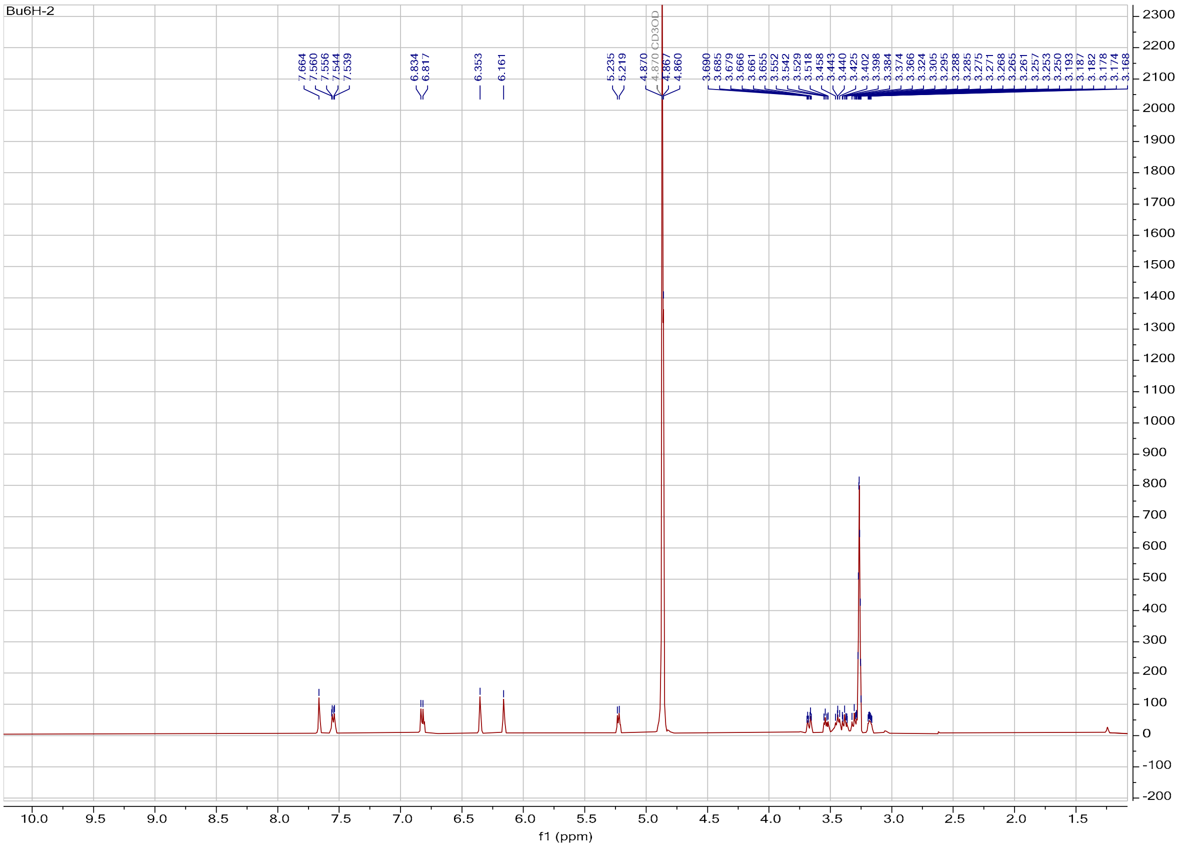

Figure S7.1. ^{1}H-NMR spectrum of compound **3** in methanol-*d*4 at 400 MHz

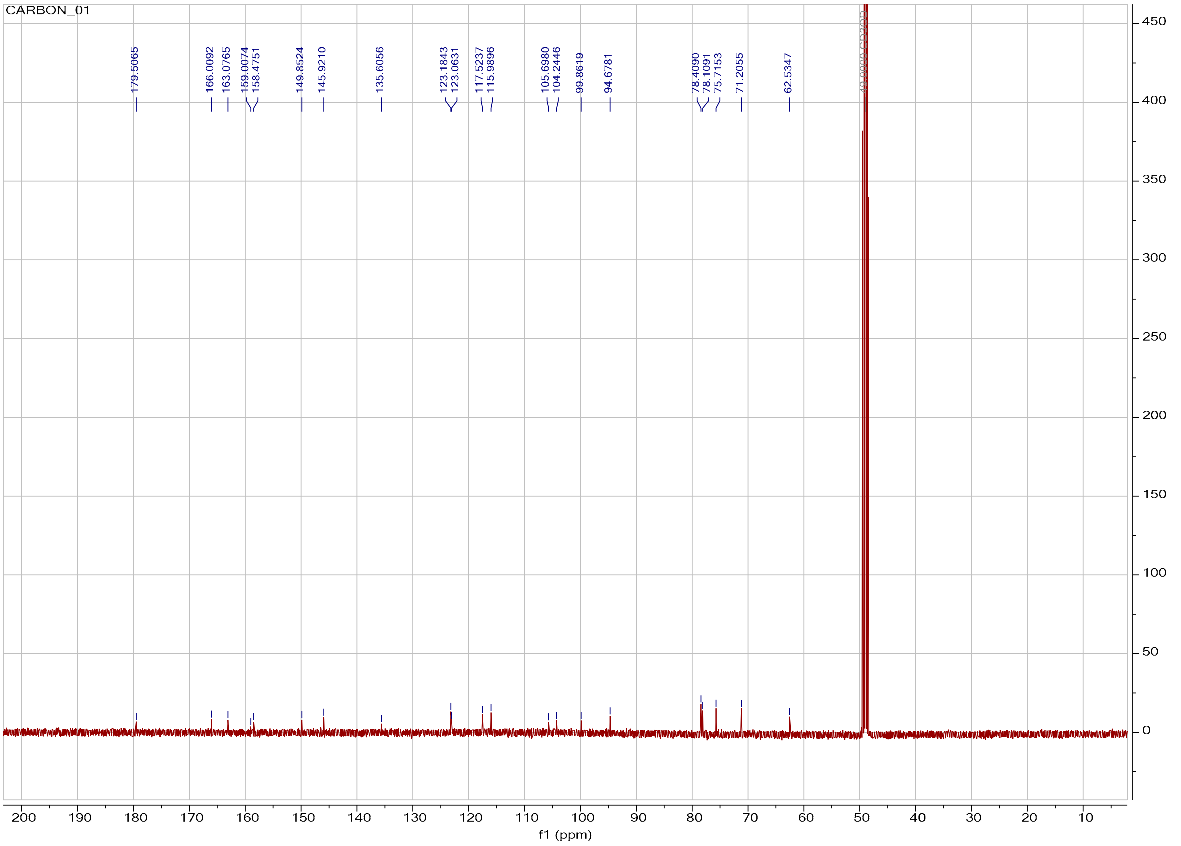

Figure S7.2. ^{13}C-NMR spectrum of compound **3** in methanol-*d*4 at 100 MHz

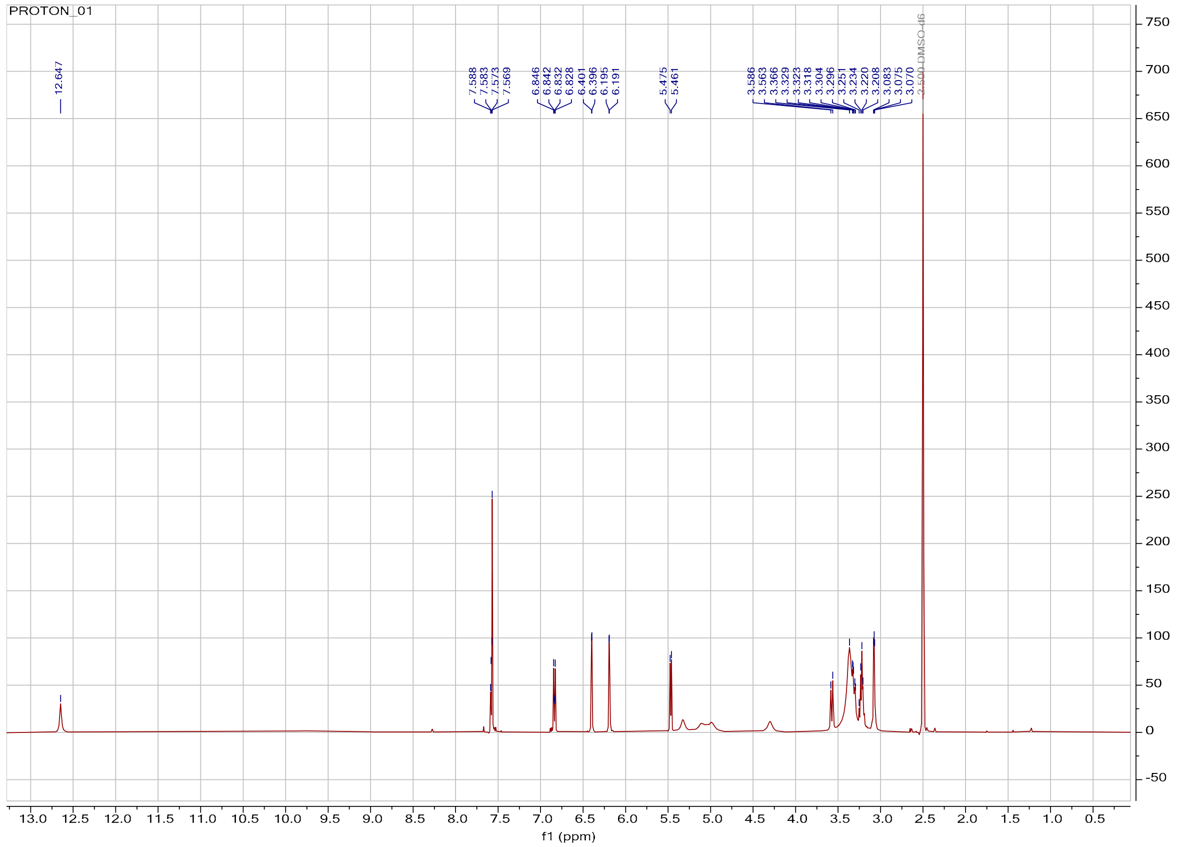

Figure S8.1. ^{1}H-NMR spectrum of compound **4** in DMSO-d_6 at 400 MHz

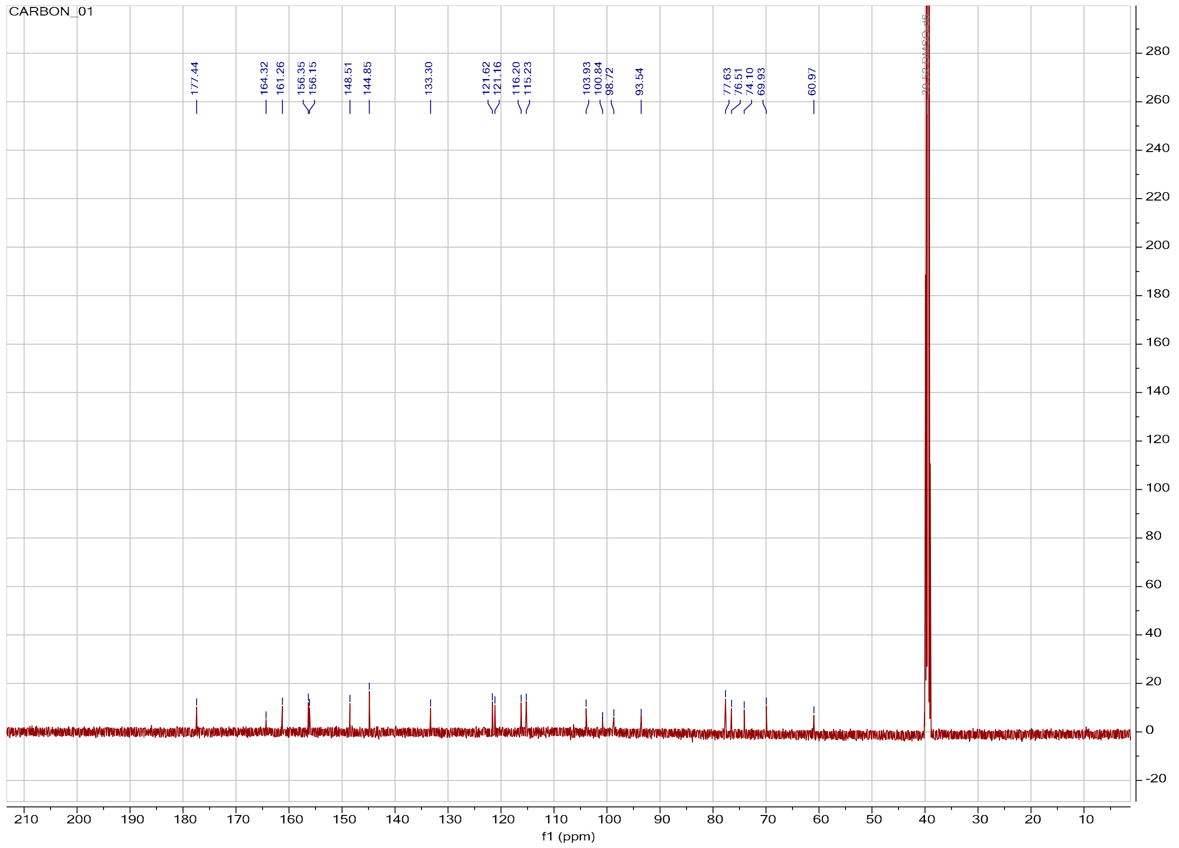

Figure S8.2. ^{13}C-NMR spectrum of compound **4** in DMSO-d_6 at 100 MHz

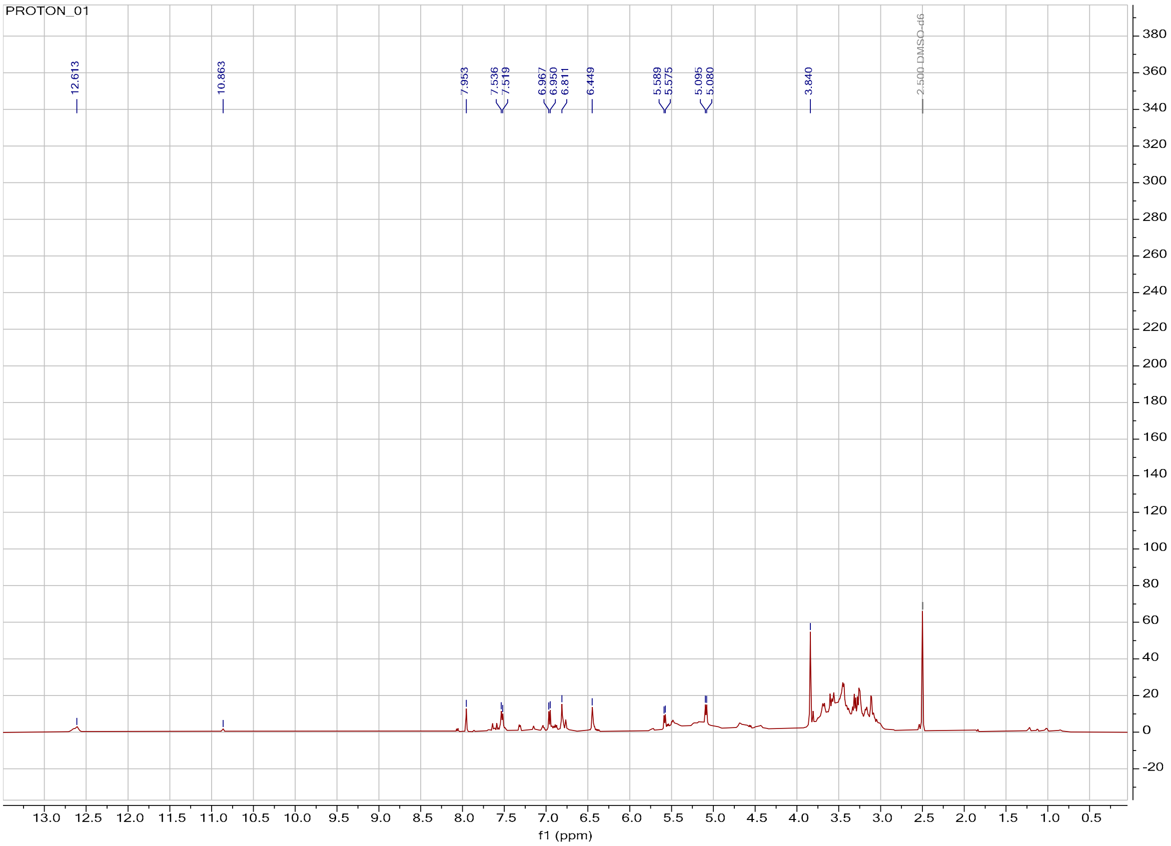

Figure S9.1. ^{1}H-NMR spectrum of compound **5** in DMSO-d_6 at 400 MHz

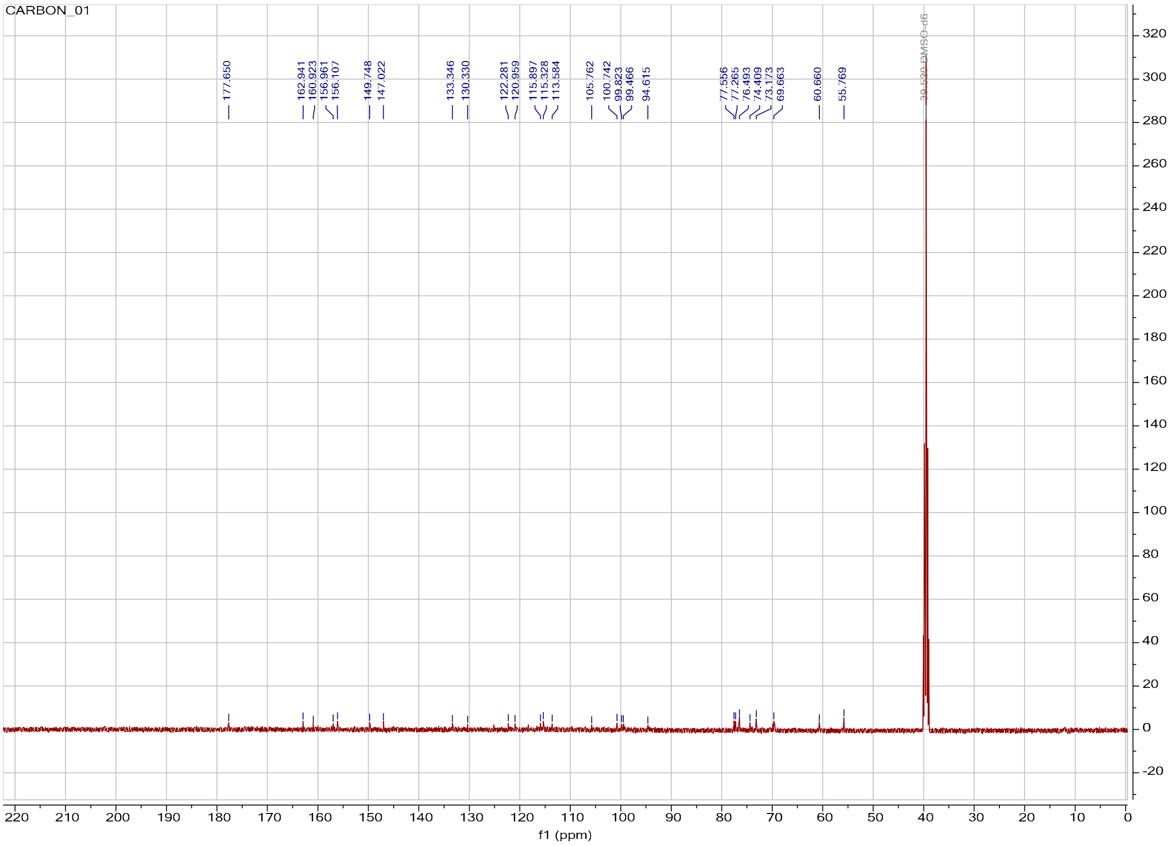

Figure S9.2. ^{13}C-NMR spectrum of compound **5** in DMSO-d_6 at 100 MHz

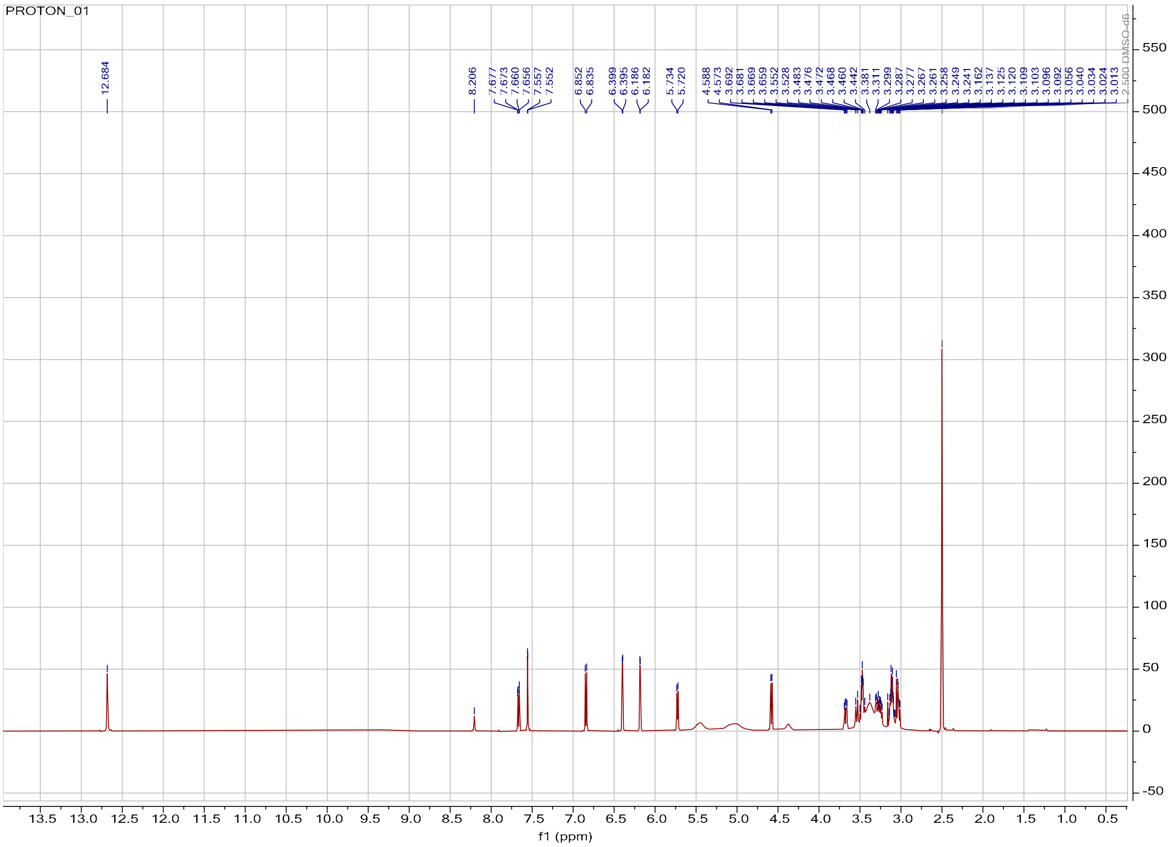

Figure S10.1. ^{1}H-NMR spectrum of compound **6** in DMSO-d_6 at 400 MHz

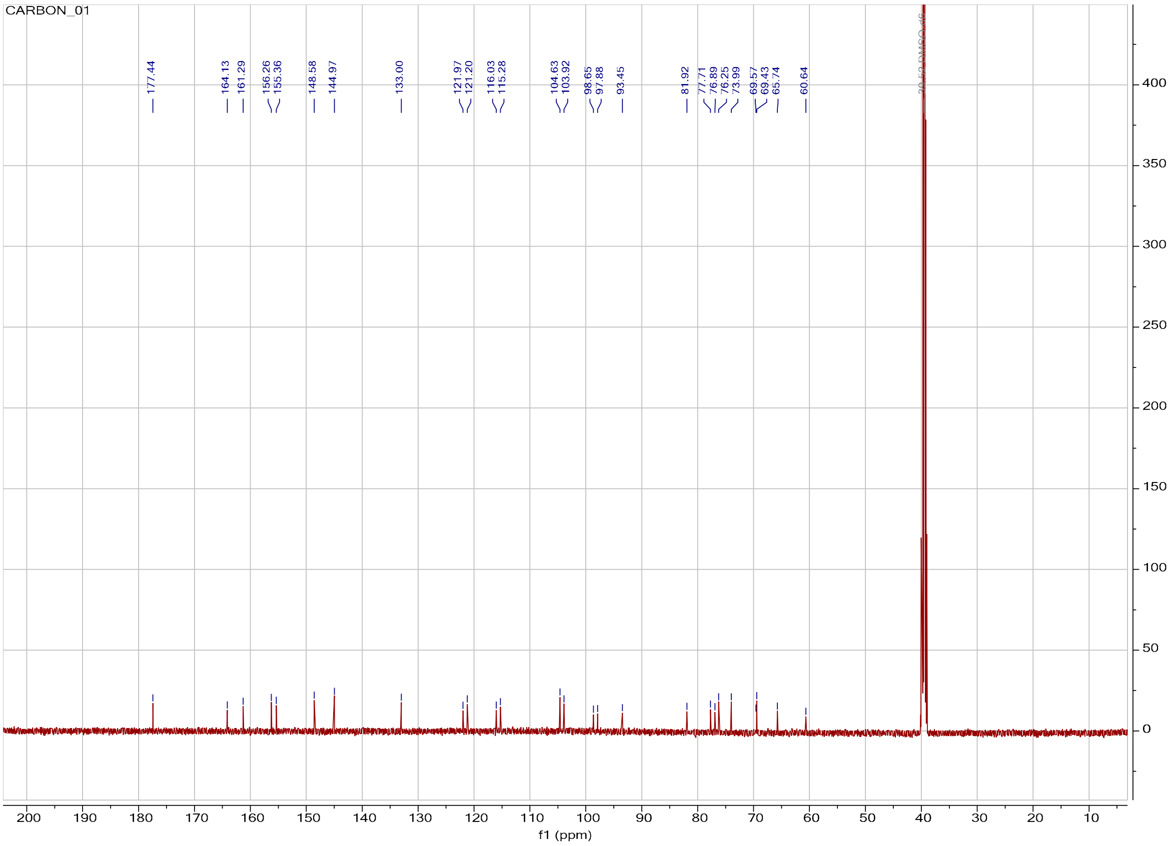

Figure S10.2. ^{13}C-NMR spectrum of compound **6** in DMSO-d_6 at 100 MHz

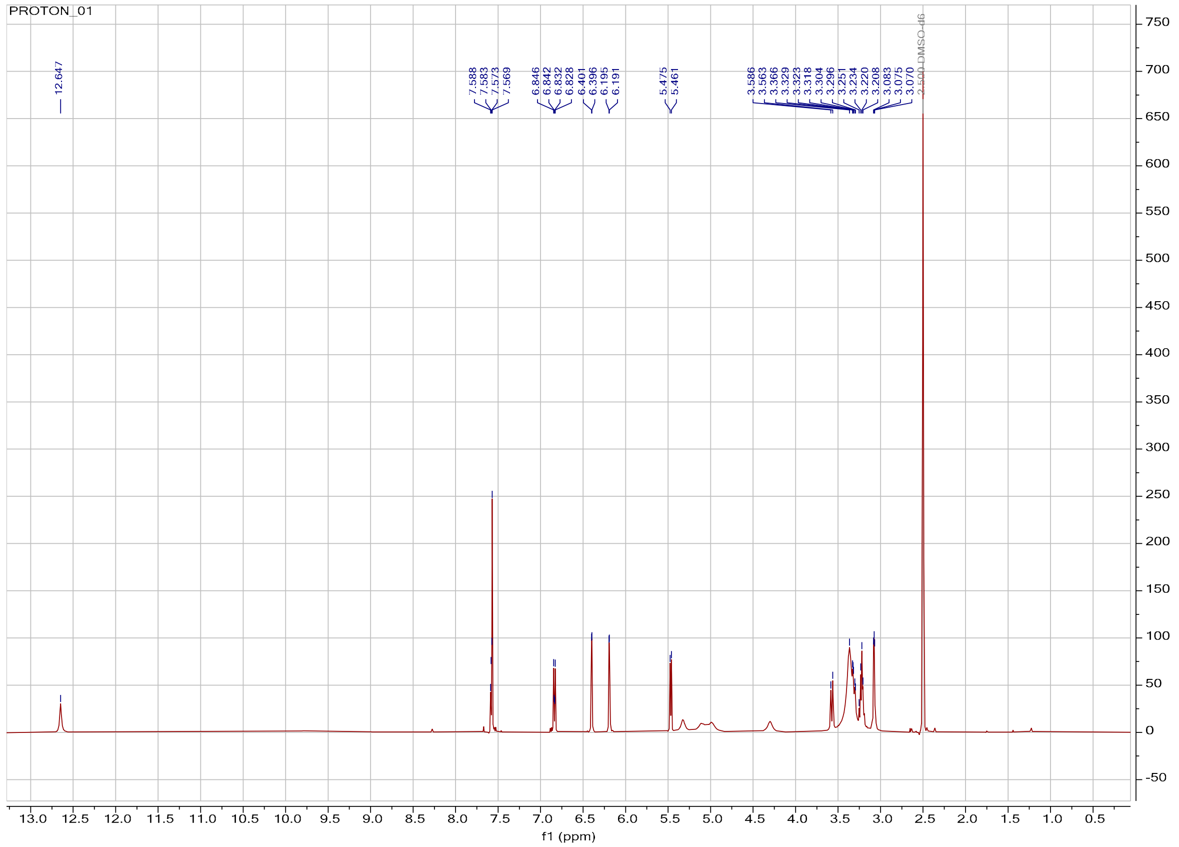

Figure S11.1. ^{1}H-NMR spectrum of compound **7** in DMSO-d_6 at 400 MHz

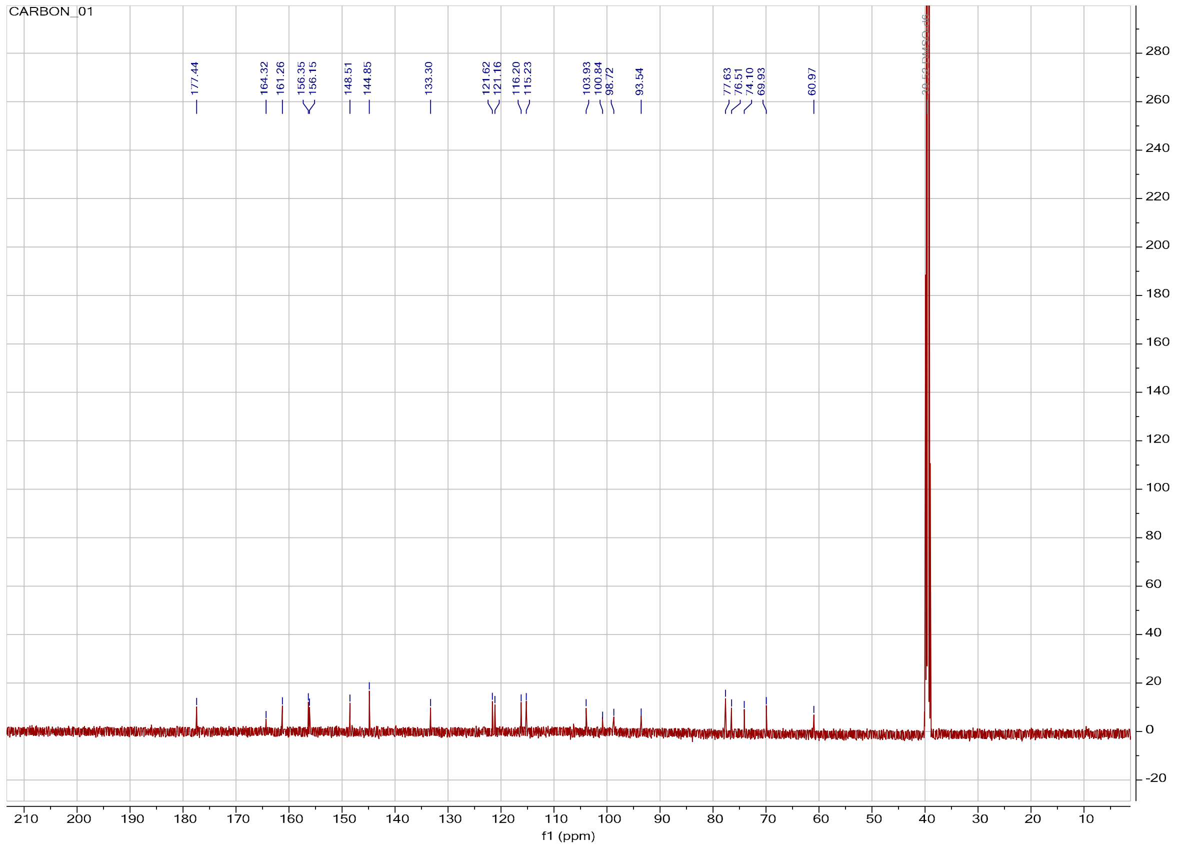

Figure S11.2. ^{13}C-NMR spectrum of compound **7** in DMSO-d_6 at 100 MHz

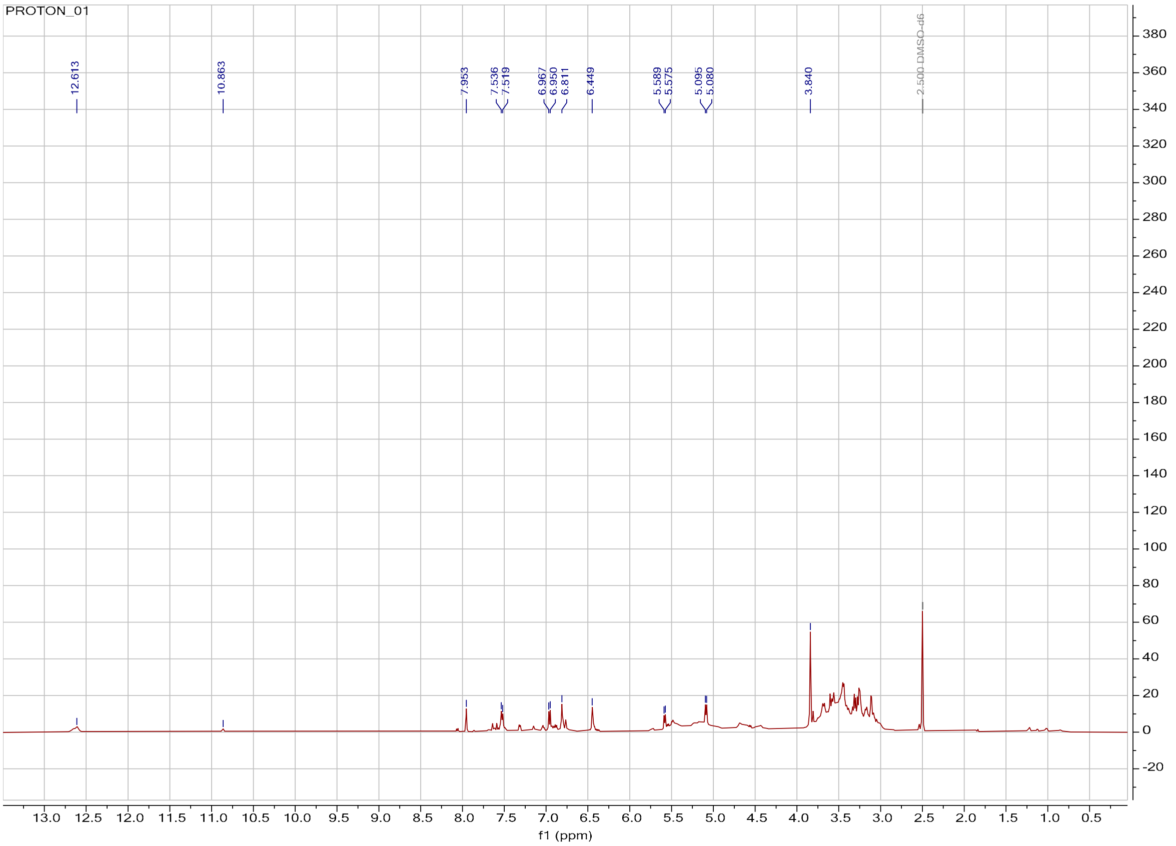

Figure S12.1. ^{1}H-NMR spectrum of compound **8** in DMSO-d_6 at 400 MHz

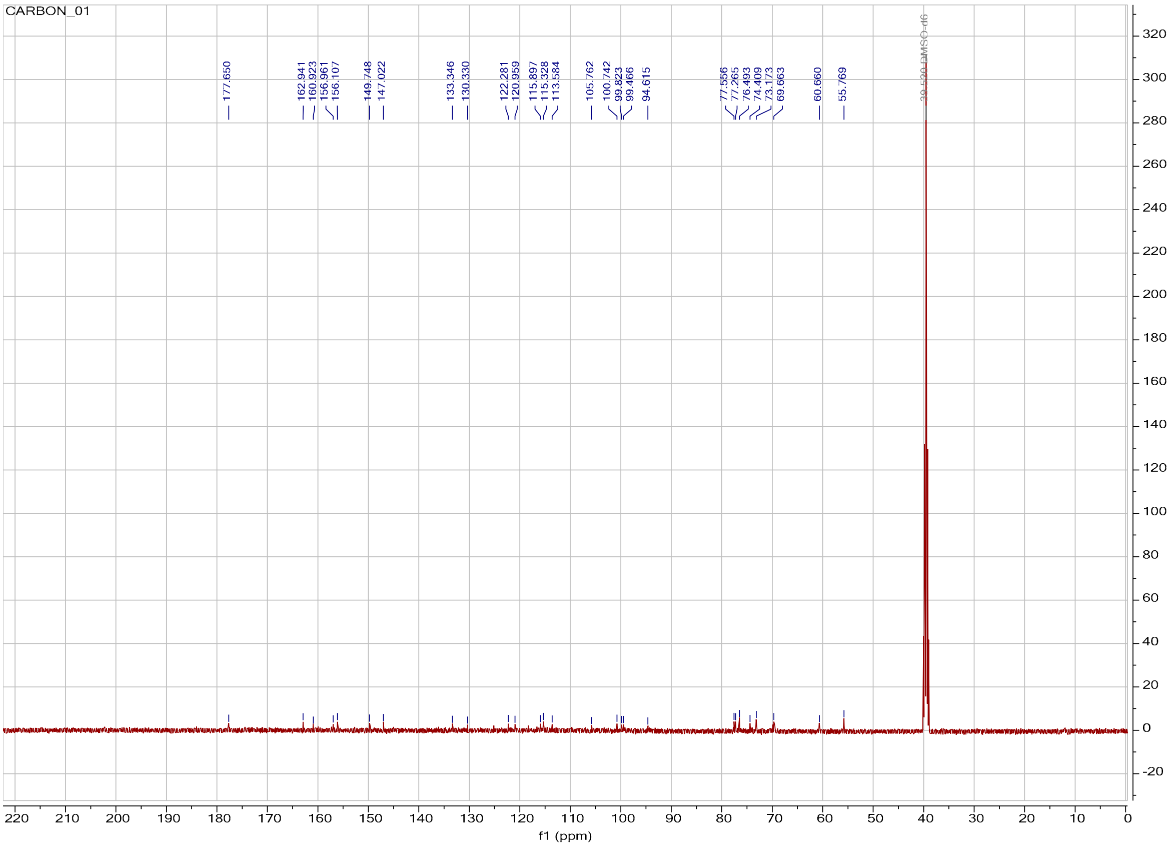

Figure S12.2. ^{13}C-NMR spectrum of compound **8** in DMSO-d_6 at 100 MHz

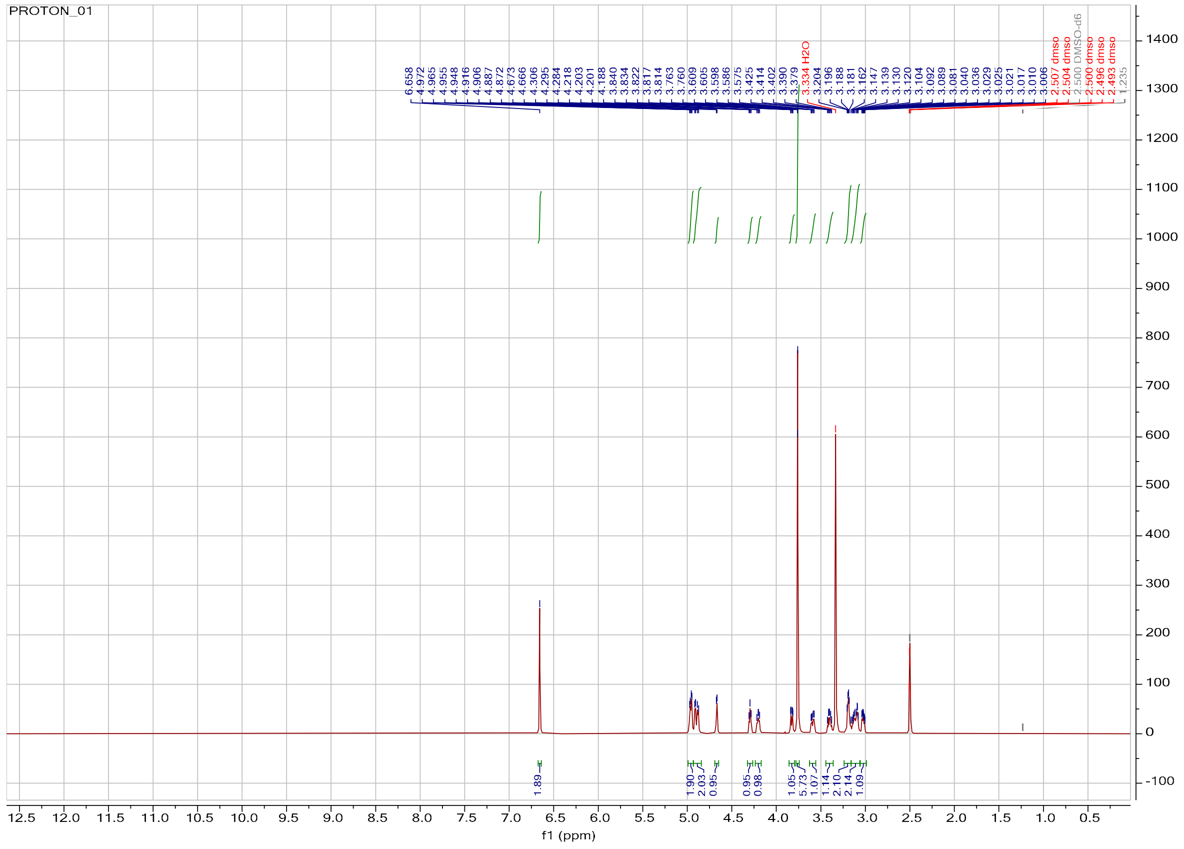

Figure S13.1. ^{1}H-NMR spectrum of compound **9** in DMSO-d_6 at 400 MHz

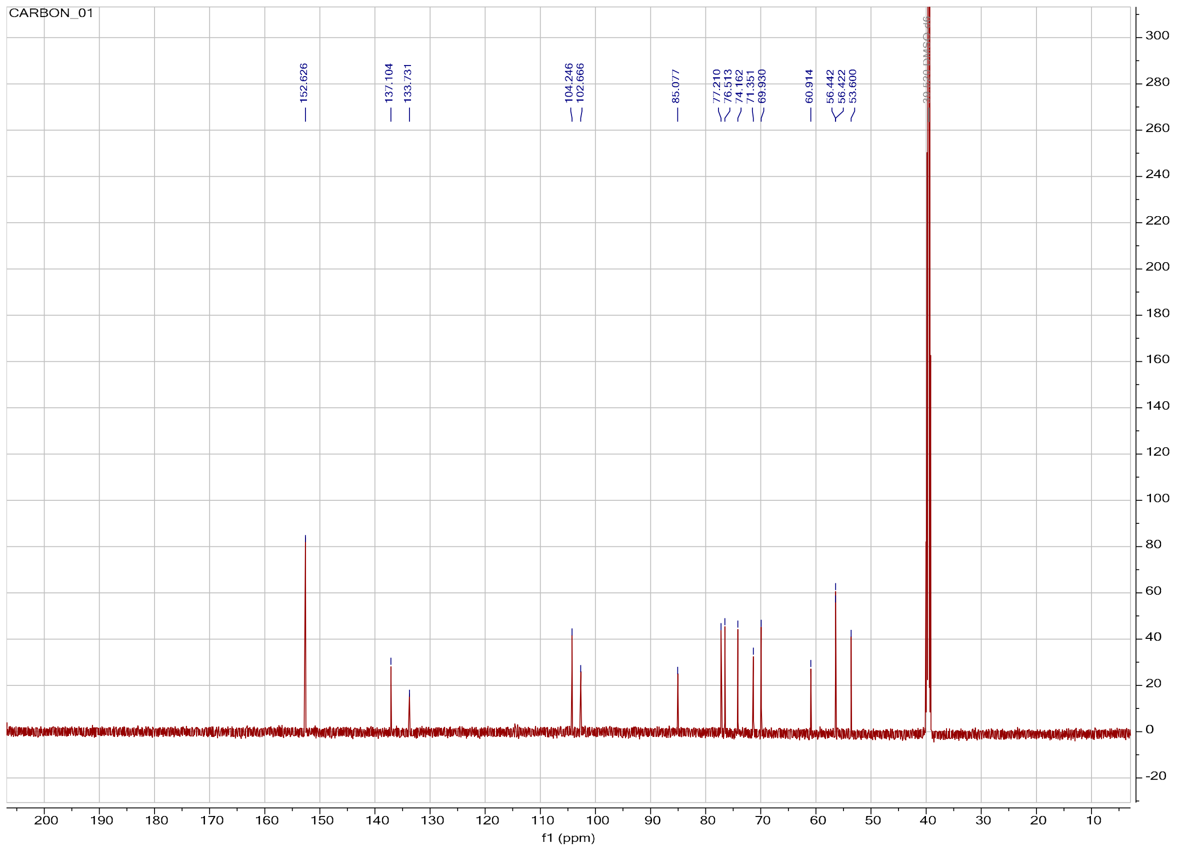

Figure S13.2. ^{13}C-NMR spectrum of compound **9** in DMSO-d_6 at 100 MHz

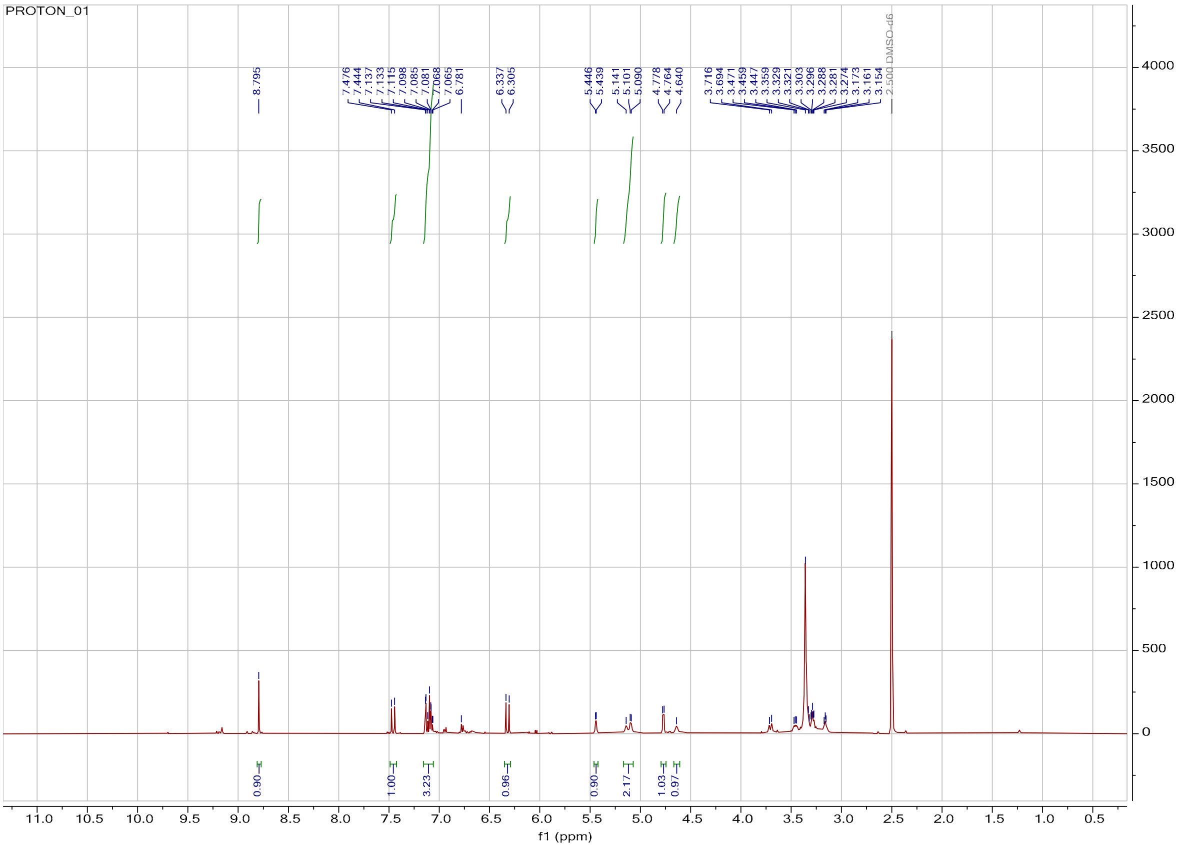

Figure S14.1. ^{1}H-NMR spectrum of compound **12** in DMSO-d_6 at 400 MHz

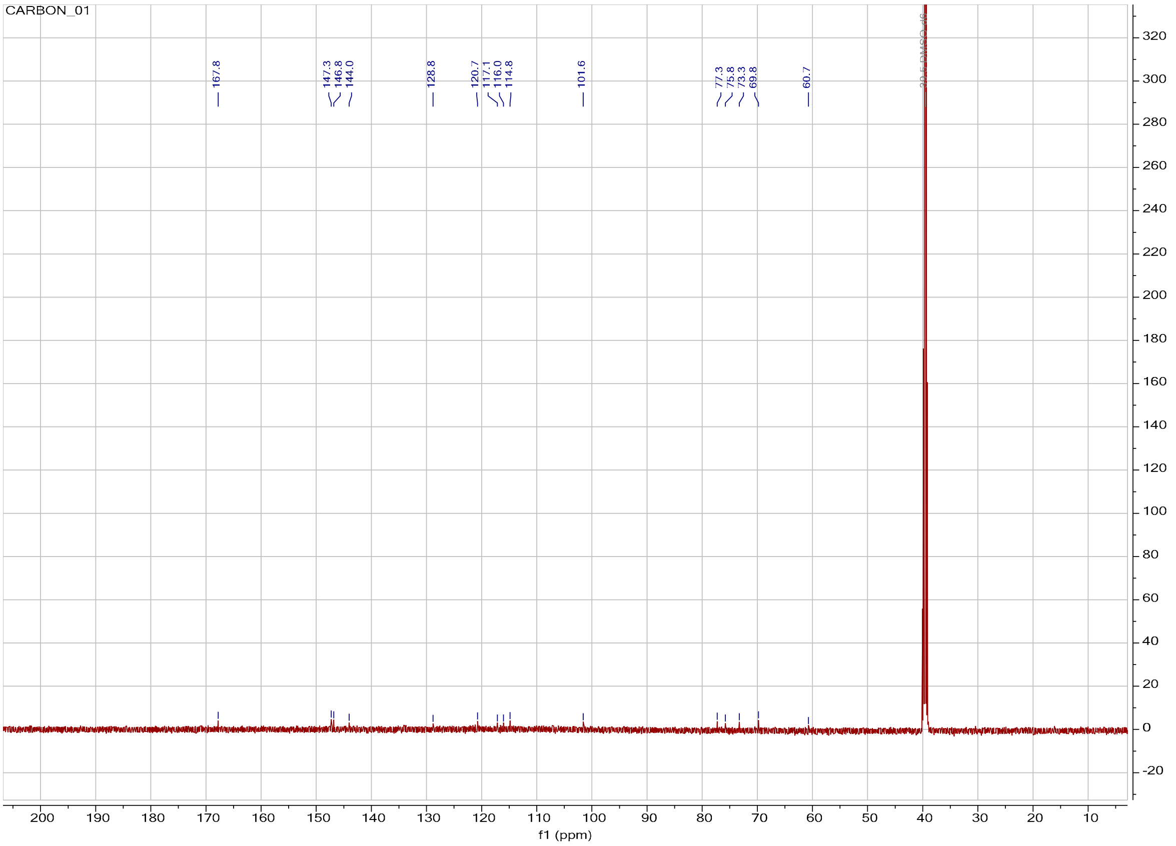

Figure S14.2. ^{13}C-NMR spectrum of compound **12** in DMSO-d_6 at 100 MHz

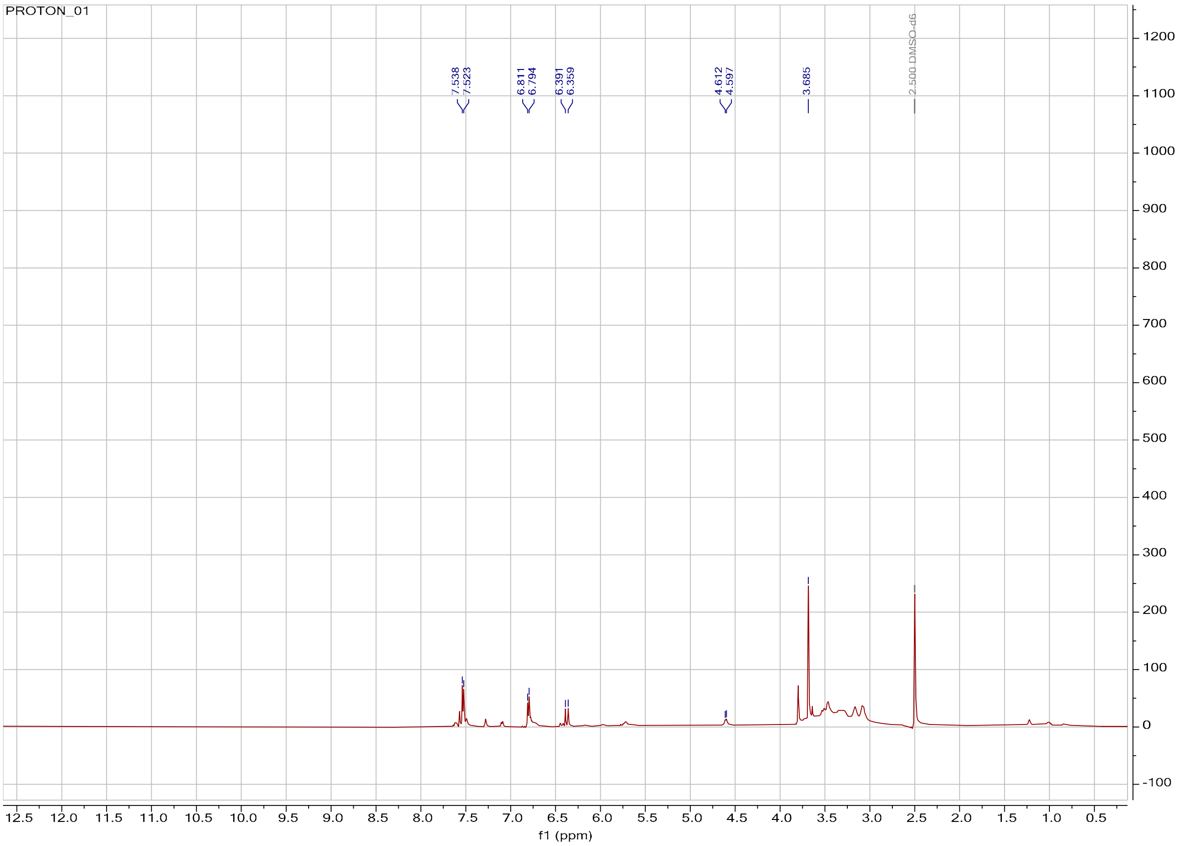

Figure S15.1. ^{1}H-NMR spectrum of compound **13** in DMSO-d_6 at 400 MHz

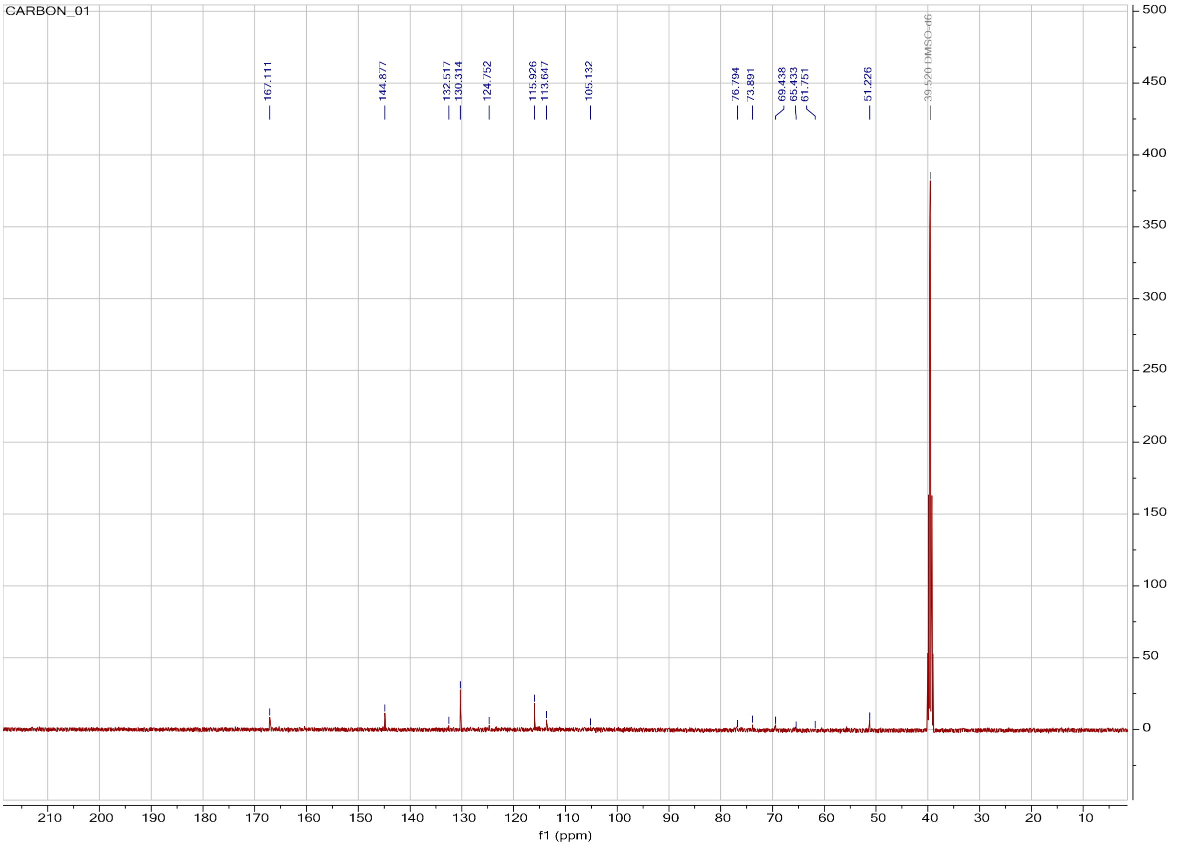

Figure S15.2. ^{13}C-NMR spectrum of compound **13** in DMSO-d_6 at 100 MHz

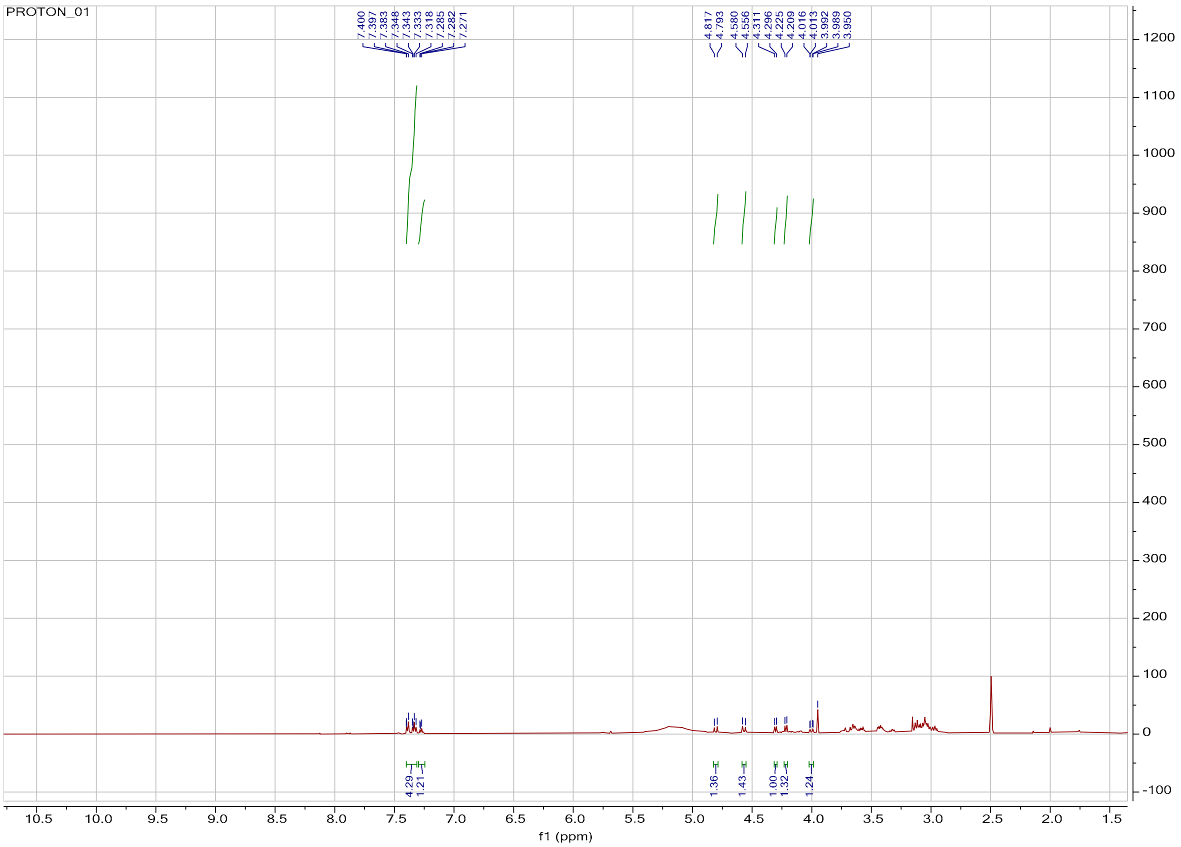

Figure S16.1. ^{1}H-NMR spectrum of compound **14** in DMSO-d_6 at 400 MHz

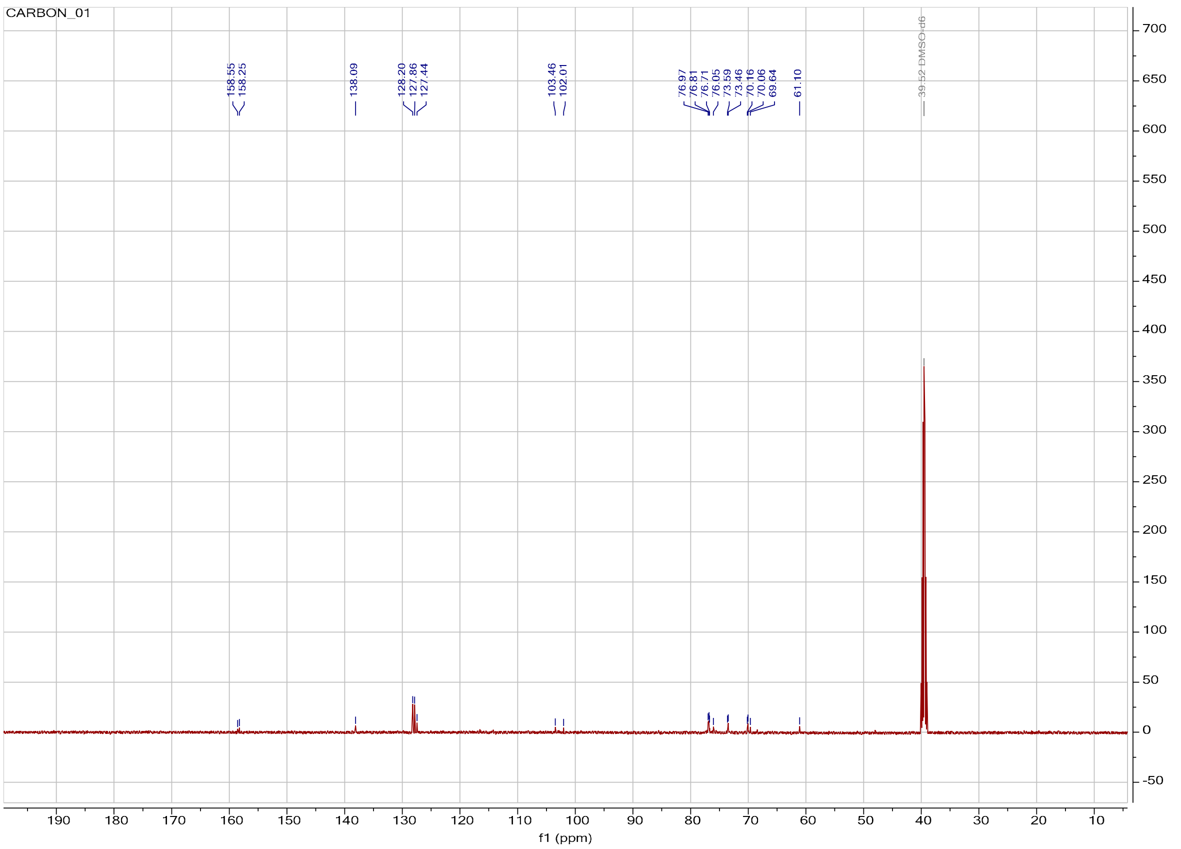

Figure S16.2. ^{13}C-NMR spectrum of compound **14** in DMSO-d_6 at 100 MHz
